# Comparative efficacy and safety of Chinese botanical drug injection in patients with sepsis: A systematic review and Bayesian network meta-analysis of randomized clinical trials

**DOI:** 10.1371/journal.pone.0343026

**Published:** 2026-03-24

**Authors:** Yue Yuan, Jiajia Wang, Siyuan Lei, Jiansheng Li

**Affiliations:** 1 Collaborative Innovation Center for Chinese Medicine and Respiratory Diseases Co-constructed by Henan Province & Education Ministry of P.R. China/Henan Key Laboratory of Chinese Medicine for Respiratory Diseases, Henan University of Chinese Medicine, Zhengzhou, China; 2 Department of Respiratory Diseases, the First Affiliated Hospital of Henan University of Chinese Medicine, Zhengzhou, China; 3 The First Clinical Medical School, Henan University of Chinese Medicine, Zhengzhou, China; Columbia University Irving Medical Center, UNITED STATES OF AMERICA

## Abstract

**Background:**

Sepsis represents a significant global health challenge, contributing to considerable morbidity, mortality, and healthcare costs. Integrating Chinese botanical drug injections (CBDIs) with Western Medical Treatments (WMT) has been increasingly recognized for its enhanced therapeutic effects in sepsis management. This Bayesian network meta-analysis aims to identify the optimal combination regimen of CBDIs and WMT for sepsis therapy.

**Methods:**

A comprehensive literature search was conducted across eight electronic databases to identify Randomized Controlled Trials (RCTs) relevant to our study criteria, spanning from their inception until January 1, 2024. The quality of included studies was rigorously assessed using the Cochrane Collaboration's Risk of Bias 2 (ROB 2) tool. Data synthesis and analysis were performed utilizing R 4.1.2 and Stata 17.0 software. Additionally, publication bias was assessed through the construction of funnel plots.

**Results:**

This network meta-analysis assessed 72 RCTs involving 6,351 participants to evaluate the effectiveness of seven CBDIs in conjunction with WMT. It found Huangqi injection to be the most effective in improving APACHE II scores. Tanreqing injections significantly reduced procalcitonin (PCT) levels, with particularly superior. Shenmai injection was most effective in decreasing C-reactive protein (CRP) levels. In terms of reducing tumor necrosis factor-alpha (TNF-α), Shenmai injection with WMT showed the best results. Xuebijing injection stood out in lowering white blood cell counts (WBC). Huangqi injection was noted for its best effectiveness in the 28-day mortality rates.

**Conclusion:**

The therapeutic efficacy of CBDIs in treating sepsis is underscored by our research findings, wherein certain botanical drugs exhibit heightened efficacy and safety attributes. The incorporation of these alternative modalities into contemporary sepsis management paradigms is advocated by the outcomes of our investigation. Nonetheless, rigorous, large-scale trials are imperative to substantiate and enhance these preliminary discoveries.

## 1. Introduction

Sepsis is a significant global health challenge, characterized by an uncontrolled immune response to infection that causes organ dysfunction and leads to considerable morbidity and mortality [1]. This complex condition imposes a heavy burden on the global healthcare system, with an estimated 31.5 million cases of sepsis and 19.4 million cases of severe sepsis annually, resulting in approximately 5.3 million deaths each year [2]. The pathophysiology of sepsis involves a critical imbalance between pro-inflammatory and anti-inflammatory responses, highlighting the urgent need for effective therapeutic interventions [3,4]. The 2018 Sepsis Treatment Guidelines emphasize the importance of the ‘Hour-1 bundle,’ advocating for prompt interventions such as lactate measurement, obtaining blood cultures prior to antibiotic administration, the use of broad-spectrum antibiotics, intravenous fluid therapy, and vasopressors within the first hour of diagnosis [5]. While these measures are effective, they have limitations, including transient benefits and the potential for adverse effects and drug resistance with prolonged use of Western medicine treatments (WMT) alone [6].

Traditional Chinese Medicine (TCM) exhibits multi-component and multi-target therapeutic properties, demonstrating unique advantages in treating complex diseases such as sepsis. Chinese botanical drug injections (CBDIs), concentrated extracts derived from herbal medicines, exert clinical efficacy largely through modulation of multiple signaling pathways. For instance, astragaloside IV from Huangqi injection and baicalin from Tanreqing injection are representative active constituents. During the early hyperinflammatory phase of sepsis, baicalin significantly reduces pro-inflammatory cytokine levels (e.g., TNF-α, IL-6) by inhibiting the TLR4/NF-κB pathway and suppresses NLRP3 inflammasome activation, thereby curtailing IL-1β release and directly intervening in cytokine storm initiation [7]. Concurrently, its ability to activate the Nrf2/HO-1 pathway neutralizes excessive ROS, mitigating oxidative stress-mediated vascular endothelial injury and alleviating sepsis-associated shock [8]. In the late immunosuppressive phase of sepsis, astragaloside IV reverses immunoparalysis by regulating the PI3K/Akt/mTOR pathway to inhibit lymphocyte hyperautophagy and apoptosis, while baicalin restores Th17/Treg balance by promoting M2 macrophage polarization and Treg cell proliferation, thereby enhancing host immune responsiveness [9,10]. These multi-target mechanisms provide a theoretical foundation for integrating CBDIs with WMT, offering a comprehensive strategy to attenuate systemic inflammatory responses and organ dysfunction observed in sepsis. The synergistic multi-target effects establish a mechanistic basis for combining CBDIs and WMT to address both inflammatory cascades and immune dysregulation in sepsis management.

In China, combining CBDIs with WMT has shown promise in treating sepsis. These adjunctive treatments have been effective in slowing disease progression, reducing mortality rates, shortening antibiotic treatment durations, and lessening adverse events [11]. CBDIs, as part of Traditional Chinese Medicine, play a significant role in treating conditions like sepsis and septic shock through a comprehensive approach targeting multiple metabolites, pathways, and targets [12]. Recognized and recommended by Chinese health authorities, these injections serve as complementary treatments, reflecting the integration of traditional and modern medical practices. Despite the variety of CBDIs available, the lack of direct comparative studies complicates the identification of the most effective CBDI regimen.

Network meta-analysis (NMA) provides a sophisticated framework for synthesizing direct and indirect evidence from clinical trials, comparing the relative efficacy of multiple interventions concurrently [13]. By employing a Bayesian approach, NMA generates probability estimates essential for clinical decision-making, facilitating the identification of superior treatment options. This study aims to rigorously assess the efficacy and safety of various CBDIs in conjunction with WMT for sepsis treatment, thereby providing robust evidence to inform the rational selection of CBDIs for managing sepsis through NMA.

## 2. Materials and methods

To ensure methodological rigor and transparency, the network meta-analysis adheres to the PRISMA-NMA guidelines [14] (S1 File). The study protocol was registered on PROSPERO (CRD42024499555) to promote transparency and reproducibility. All plant species were taxonomically validated using the Plants of the World Online database [15]. We included complete species names, authorities, family affiliations, and any assigned drug names according to pharmacopeial standards. Detailed descriptions of metabolites for all included Chinese botanical drug injections are provided in S2 File. To comprehensively evaluate the safety of each CBDI, we referred to the respective product inserts and summarized their side effects, interactions, and general safety in S2 File. Patients or the public were not involved in the design, or conduct, or reporting, or dissemination plans of our research.

### 2.1. Literature search

We systematically searched eight electronic databases: PubMed, Embase, Web of Science, Cochrane Library, China National Knowledge Infrastructure (CNKI), Wanfang Database, China Science and Technology Journal Database (VIP), and Chinese Biomedical Literature Database (CBM) up to January 1, 2024. Searches combined MeSH terms with free-text terms focusing on “Sepsis,” “Injection,” and “Randomized Controlled Trials (RCTs)” without language restrictions. The search strategy was tailored to each database based on their unique characteristics and the PICO principle (S3 File). Validated search filters with high sensitivity for randomised controlled trials (RCTs) were applied for searching MEDLINE and EMBASE [16,17]. Manual searches of reference lists of relevant articles complemented the electronic search.

### 2.2. Inclusion criteria

#### 2.2.1. Participants.

The study included adults (≥18 years) diagnosed with sepsis, adhering to specific accepted diagnostic criteria. Patients included in the study were not restricted from complications other than sepsis. There were no limitations or exclusions based on gender, race, or nationality.

#### 2.2.2. Interventions.

The experimental group received CBDIs alongside standard WMT, without restrictions on treatment duration.

#### 2.2.3. Comparisons.

The control group received standard WMT alone, including fluid resuscitation, anti-infective therapy, and vasoactive drugs. There were no limitations or exclusions based on the use of placebo.

#### 2.2.4. Outcomes.

Primary outcome was Acute Physiology and Chronic Health Evaluation (APACHE II scores), with secondary outcomes including procalcitonin (PCT), C-reactive protein (CRP), tumor necrosis factor-alpha (TNF-α), white blood cell (WBC), and 28-day mortality.

#### 2.2.5. Types of studies.

Only RCTs assessing CBDIs for sepsis treatment were included, with a prerequisite of at least 2 study per CBDI.

### 2.3. Exclusion criteria

Studies with either of the following criterion were excluded: (1) Duplicates; (2) Non-RCT studies; (3) Review articles (review papers, systematic review or meta-analysis); (4) Animal tests.

### 2.4. Literature screening and data extraction

Two researchers independently screened studies and extracted data, with disagreements resolved by consultation with a third researcher. EndNote X9 managed the literature, and IBM SPSS Statistics 25 facilitated data extraction, which included study characteristics, participant demographics, intervention details, and outcomes. Discrepancies were resolved through consensus or third-party adjudication.

### 2.5. Quality assessment

The Cochrane Collaboration's Risk of Bias 2.0 tool assessed the risk of bias across several domains [18]. Studies were rated as low risk, some concern, or high risk. A high overall risk of bias was assigned if any domain was rated high, and unclear if two or more domains were uncertain.

### 2.6. Statistical analysis

R software (version 4.1.2) performed the network meta-analysis using the “gemtc” package, employing the Markov chain Monte Carlo method. The analysis differentiated between dichotomous (odds ratio, OR) and continuous outcomes (mean differences, MD, or standardized mean differences, SMD). Network group commands processed data, and indirect evidence was visualized. The SUCRA was calculated for treatment ranking. Publication bias was evaluated through funnel plot asymmetry in Stata (version 17.0). The “mtc.anohe” function was conducted to test the homogeneity hypothesis. Statistical significance was determined when the 95% credible interval (CI) did not include the null value (zero for mean differences and one for odds ratios), corresponding to a posterior probability <0.05. No additional correction for multiple testing (such as FDR adjustment) was applied, as the Bayesian framework inherently accounts for uncertainty in the posterior estimates. Additionally, a subgroup network meta-analysis was conducted, considering the potential differences between sepsis and septic shock, to address the heterogeneity and ensure the robustness of the results.

### 2.7. Evaluation of the evidence

The quality of evidence for outcomes was evaluated using the GRADE methodology [19]. Initially, all included RCTs were considered high-quality evidence. However, five factors could lead to a downgrading of the evidence quality: risk of bias (such as inadequate allocation concealment and lack of blinding), inconsistency (narrow or no overlap in confidence intervals across studies, large I² values), indirectness (differences in population, intervention, or outcome measures), imprecision (small sample sizes, excessively wide confidence intervals for effect size estimates), and publication bias (potential bias when studies are funded by vendors). Based on these factors, the quality of evidence was categorized as high (no downgrading), moderate (one level downgrade), low (two levels downgrade), or very low (three levels downgrade).

## 3. Results

### 3.1. Results of the search

**Fig 1:**
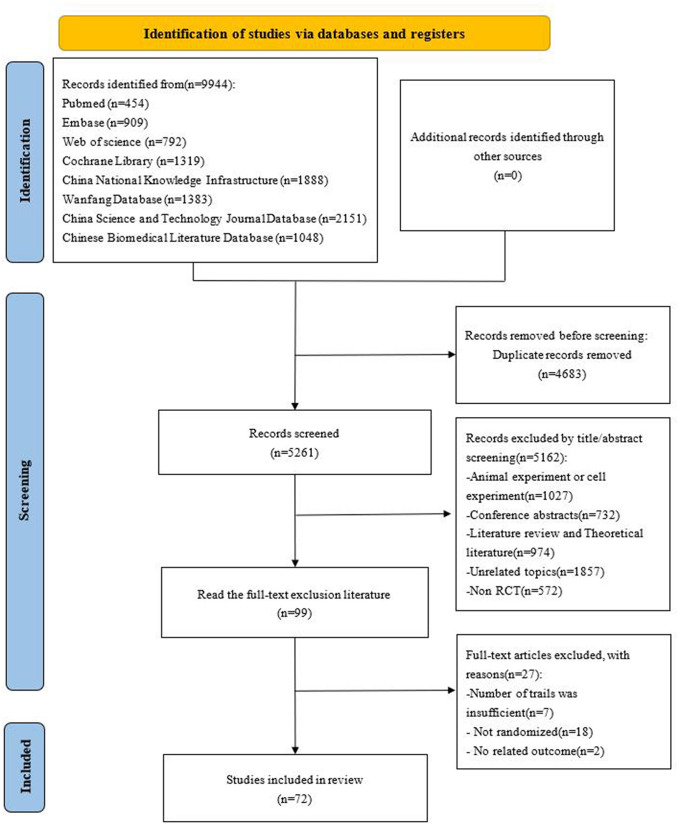
Flow diagram of study inclusion.

The search strategy initially yielded 9,944 studies. After removing duplicates, 5,261 studies remained. Title and abstract screening excluded 5,162 studies, leaving 99 for full-text review to determine their suitability for inclusion. Ultimately, 72 studies met the criteria and were incorporated into the analysis. This literature selection process is detailed in Fig 1.

This figure summarizes the literature search and study selection process in accordance with the PRISMA guidelines. It presents the numbers of records identified through database searching, duplicates removed, studies screened by title and abstract, full-text articles assessed for eligibility, and randomized controlled trials ultimately included in the network meta-analysis.

### 3.2. Study characteristics

**Fig 2:**
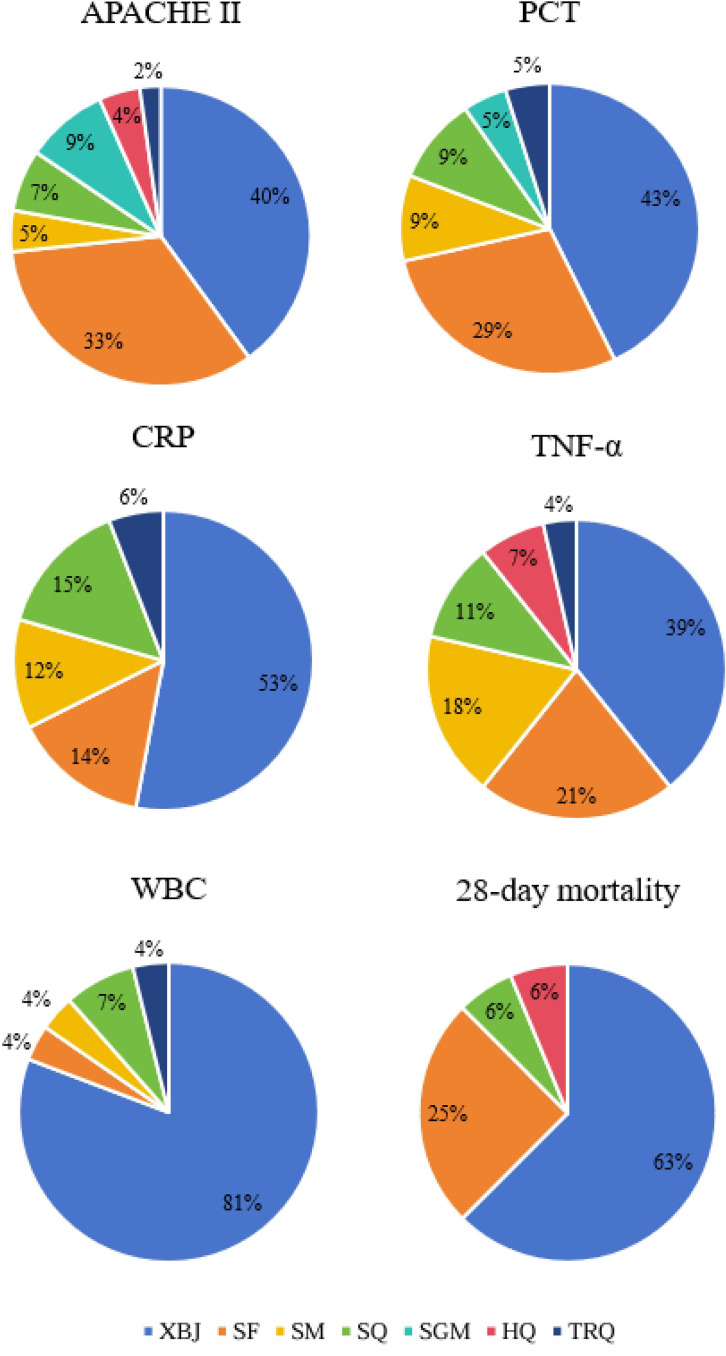
Distribution of randomized controlled trials across different CBDIs.

The NMA incorporated 72 RCTs [20–91], covering 6,351 patients, with study sizes varying from 30 to 1,817 participants. These were all dual-arm trials, with control groups receiving WMT as standard care. The interventions in the treatment groups involved one of the CBDIs, specifically: Xuebijing injection (XBJ, n = 37), Shenfu injection (SF, n = 17), Shenmai injection (SM, n = 5), Shenqifuzheng injection (SQ, n = 5), Shengmai injection (SGM, n = 4), Huangqi injection (HQ, n = 2), and Tanreqing injection (TRQ, n = 2). The main characteristics of the included studies are summarized in S4 File. Given that the evidence network did not include any direct comparisons between different CBDIs and consisted exclusively of dual-arm trials comparing CBDIs plus WMT with WMT alone, a pie chart was used to illustrate the distribution of studies across interventions. Fig 2 presents the distribution of included trials across different CBDIs, reflecting the proportion of studies contributing evidence to each intervention.

The pie chart illustrates the proportion of included randomized controlled trials (RCTs) evaluating each CBDI in combination with Western medicine treatment (WMT). The proportions are calculated based on the total number of included trials (n = 72), with each segment representing the relative contribution of studies investigating a specific CBDI plus WMT versus WMT alone. This figure reflects the distribution of available evidence across interventions.

Abbreviations: CBDI, Chinese botanical drug injection; WMT, Western medicine treatment; XBJ, Xuebijing injection; SF, Shenfu injection; SM, Shenmai injection; SQ, Shenqifuzheng injection; SGM, Shengmai injection; HQ, Huangqi injection; TRQ, Tanreqing injection.

### 3.3. Quality assessment

**Fig 3:**
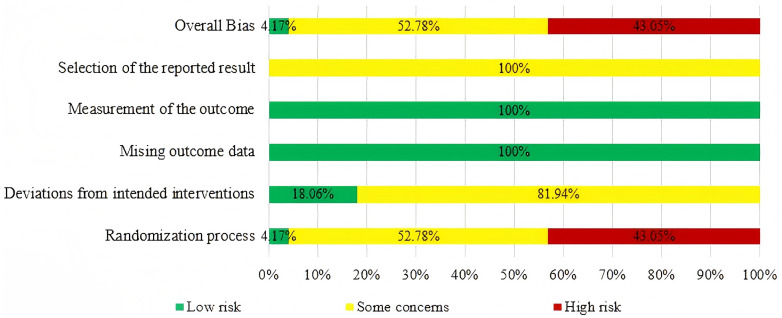
The risk of bias assessment.

Quality assessment was performed to evaluate the methodological rigor and risk of bias of the included RCTs, ensuring that the reliability and validity of the network meta-analysis results could be appropriately interpreted. The quality of the included studies was evaluated using the Revised Cochrane Risk-of-Bias Tool for Randomized Trials (RoB.2). Thirty-one RCTs were assessed as “high risk” due to their exclusive mention of “random” without explicit elucidation of the methodology employed for generating random sequences. Thirty-eight RCTs were classified as presenting “some concerns” because, despite evident random sequence generation, they failed to demonstrate allocation concealment. Consequently, merely three RCTs were designated as “low risk” attributable to the precise methods utilized for both random sequence generation and allocation concealment. The risk of bias analysis points to 59 RCTs of poorly or non-reported aspects in blinding allocation concealment. Three RCTs reported blinding, and 11 of them were trials of single-blind methods, the use of blinding was not reported in the remaining 59 trials. Thus, 69 RCTs, was rated as having “some concerns” regarding deviations from intended interventions. The included RCTs did not report any cases of patient loss, thus all the included RCTs were assessed as having a “low risk” rating. The included RCTs did not have access to pre-designed protocols, resulting in a rating of “some concerns” for selection of reported result. Overall, three studies were classified as low risk of bias, 39 studies as having some concerns, and 31 studies as high risk of bias. Detailed judgments for each RoB 2.0 domain are provided in S5 File, and the overall risk-of-bias assessment is summarized in Fig 3. Importantly, the risk-of-bias assessments were not incorporated as quantitative weighting factors in the network meta-analysis models; instead, they were used qualitatively to inform the interpretation of results and to support the certainty-of-evidence evaluation within the GRADE framework.

Risk of bias was evaluated using the RoB 2.0. The figure summarizes judgments across the five methodological domains: randomization process, deviations from intended interventions, missing outcome data, measurement of the outcome, and selection of the reported result, as well as the overall risk-of-bias judgment for each included study.

### 3.4. Network meta-analysis

#### 3.4.1. Primary outcome:.

**Fig 4:**
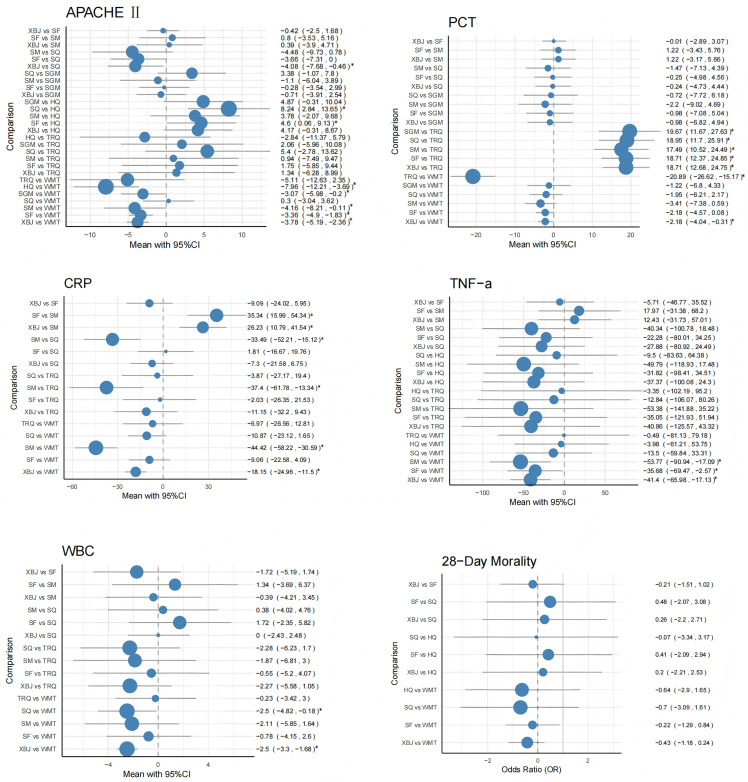
Forest plot of the comparative effects of CBDIs on outcomes.

**APACHE Ⅱ score** APACHE II score is a validated tool for assessing disease severity in critically ill patients; higher scores indicate worse prognosis in sepsis, so it was chosen to evaluate the impact of CBDIs on illness severity. Across 45 RCTs involving seven different CBDIs, most interventions demonstrated reductions in APACHE II scores compared with WMT alone, indicating a potential benefit in alleviating disease severity in sepsis. Specifically, XBJ, SF, SM, SGM, and HQ combined with WMT were associated with significant score improvements, with HQ showing the most pronounced reduction (MD = –7.96, 95% CI: –12.21 to –3.69), SM also demonstrating a notable effect (MD = –4.16, 95% CI: –8.21 to –0.11), followed by XBJ (MD = –3.78, 95% CI: –5.19 to –2.36), SF (MD = –3.36, 95% CI: –4.9 to –1.83), and SGM (MD = –3.07, 95% CI: –5.98 to –0.2), while SQ and TRQ did not display consistent advantages. In network comparisons, XBJ outperformed SQ (MD = –4.08, 95% CI: –7.68 to –0.46), and HQ was superior to both SF (MD = 4.6, 95% CI: 0.06 to 9.13) and SQ (MD = 8.24, 95% CI: 2.84 to 13.65), suggesting differential efficacy among CBDIs. Overall, HQ showed the largest numerical reduction, XBJ provided consistent and stable benefits, SM demonstrated favorable effects though with some heterogeneity, SF and SGM yielded moderate but clinically meaningful improvements, whereas SQ and TRQ showed little or no additional benefit beyond WMT. The comparative efficacy of these interventions is detailed in Fig 4.

#### 3.4.2. Secondary outcome.

**PCT** PCT is a sensitive biomarker of bacterial infection and sepsis severity, thus included to assess the anti-infective efficacy of CBDIs. Across 21 RCTs involving seven CBDIs, significant reductions in PCT levels were observed when XBJ and TRQ were used alongside WMT, compared with WMT alone. Specifically, XBJ + WMT reduced PCT levels by MD = –2.18 (95% CI: –4.04 to –0.31), and TRQ + WMT achieved a markedly larger reduction of MD = –20.89 (95% CI: –26.62 to –15.17); however, the number of trials evaluating TRQ was relatively small, which may limit the robustness of this result. Network comparisons further highlighted the differential efficacy among CBDIs, with TRQ + WMT showing the most substantial effect: XBJ + WMT vs. TRQ + WMT (MD = 18.71, 95% CI: 12.68 to 24.85), SF + WMT vs. TRQ + WMT (MD = 18.71, 95% CI: 12.37 to 24.85), SM + WMT vs. TRQ + WMT (MD = 17.49, 95% CI: 10.52 to 24.49), SQ + WMT vs. TRQ + WMT (MD = 18.95, 95% CI: 11.7 to 25.91), and SGM + WMT vs. TRQ + WMT (MD = 19.67, 95% CI: 11.67 to 27.63). Overall, TRQ + WMT demonstrated the largest PCT reduction, XBJ + WMT provided moderate but significant improvement, whereas SF + WMT (MD = –0.39, 95% CI: –3.9 to 4.71), SM + WMT (MD = –0.3, 95% CI: –3.04 to 3.62), SQ + WMT (MD = –0.28, 95% CI: –3.54 to 2.99), and SGM + WMT (MD = –3.07, 95% CI: –5.98 to –0.2) did not show consistent significant reductions in PCT levels. The detailed comparisons of these interventions are outlined in Fig 4.

**CRP** CRP reflects systemic inflammation, and persistently high levels predict poor outcomes, making it a useful marker for evaluating the anti-inflammatory effects of CBDIs. Across 34 RCTs involving five CBDIs, the addition of XBJ and SM to WMT was notably more effective in reducing CRP levels compared with WMT alone. Specifically, XBJ + WMT reduced CRP by MD = –18.15 (95% CI: –24.96 to –11.5), and SM + WMT by MD = –44.42 (95% CI: –58.22 to –30.59). Network comparisons further highlighted differential efficacy among CBDIs: XBJ + WMT vs. SM + WMT (MD = 26.23, 95% CI: 10.79 to 41.54), SF + WMT vs. SM + WMT (MD = 35.34, 95% CI: 15.99 to 54.34), SM + WMT vs. SQ + WMT (MD = –33.49, 95% CI: –52.21 to –15.12), and SM + WMT vs. TRQ + WMT (MD = –37.4, 95% CI: –61.78 to –13.34). Overall, SM + WMT demonstrated the largest CRP reduction, XBJ + WMT also provided significant improvement, whereas SF + WMT (MD = 1.81, 95% CI: –16.67 to 19.76), SQ + WMT (MD = –3.87, 95% CI: –27.17 to 19.4), and TRQ + WMT (MD = –6.97, 95% CI: –26.56 to 12.81) did not show consistent significant reductions in CRP levels. These comparisons among different interventions are detailed in Fig 4.

**TNF-α** TNF-α is a key pro-inflammatory cytokine in sepsis pathogenesis, and measuring it helps determine whether CBDIs modulate excessive immune responses. Across 28 studies evaluating treatments, XBJ, SF, and SM combined with WMT significantly reduced TNF-α levels compared with WMT alone. Specifically, XBJ + WMT (MD = –41.4, 95% CI: –65.98 to –17.13), SF + WMT (MD = –35.68, 95% CI: –69.47 to –2.57), and SM + WMT (MD = –53.77, 95% CI: –90.94 to –17.09) showed significant reductions. Network comparisons did not indicate that any one injection was significantly superior to another, suggesting that these CBDIs have comparable efficacy in modulating TNF-α levels. Overall, SM + WMT demonstrated the largest reduction in TNF-α, followed by XBJ + WMT and SF + WMT, whereas SQ + WMT (MD = –9.5, 95% CI: –83.63 to 64.38), HQ + WMT (MD = –3.35, 95% CI: –102.19 to 95.2), and TRQ + WMT (MD = –0.49, 95% CI: –81.13 to 79.18) did not show consistent significant reductions compared with WMT alone. These findings, illustrating the comparative effectiveness of different interventions, are detailed in Fig 4.

**WBC** WBC is a key hematological parameter reflecting systemic inflammatory activity and immune response, and its measurement is important for evaluating the therapeutic effects of CBDIs in patients with sepsis. Across 26 studies evaluating treatments, XBJ + WMT (MD = –2.5, 95% CI: –3.3 to –1.68) and SQ + WMT (MD = –2.5, 95% CI: –4.82 to –0.18) showed significant reductions in WBC levels compared with WMT alone. Network comparisons did not indicate that any one injection was significantly superior to another, suggesting that these CBDIs have comparable efficacy in modulating WBC levels. Other interventions, including SF + WMT (MD = 1.34, 95% CI: –3.69 to 6.37), SM + WMT (MD = 1.72, 95% CI: –2.35 to 5.82), and TRQ + WMT (MD = –0.23, 95% CI: –3.42 to 3.0), did not show consistent significant reductions compared with WMT alone. These interventions’ comparative results are detailed in Fig 4.

**28-day mortality** 28-day mortality is a standard clinical endpoint in sepsis trials and was used to evaluate the survival benefit of CBDIs. Across 16 studies investigating four CBDIs, all interventions showed a trend toward reduced 28-day mortality compared to WMT, although none reached statistical significance. Specifically, HQ + WMT showed a log odds ratio (LOR) of –0.43 (95% CrI: –1.18, 0.24), SF + WMT: –0.22 (95% CrI: –1.29, 0.84), XBJ + WMT: –0.43 (95% CrI: –1.18, 0.24), and SQ + WMT: –0.21 (95% CrI: –1.51, 1.02). Network comparisons among the CBDIs did not reveal any clear superiority, with overlapping credible intervals, indicating comparable effects on 28-day mortality across the interventions. Overall, all CBDIs consistently trend toward reducing mortality, but current evidence is insufficient to demonstrate statistically significant benefit for any particular injection. Detailed comparisons of these interventions are outlined in Fig 4.

The forest plots display pooled effect estimates derived from the Bayesian network meta-analysis for APACHE II score, procalcitonin (PCT), C-reactive protein (CRP), tumor necrosis factor-alpha (TNF-α), white blood cell count (WBC), and 28-day mortality. For continuous outcomes, results are presented as mean differences (MDs) with 95% credible intervals (CrIs), whereas for dichotomous outcomes, effect estimates are expressed as odds ratios (ORs) with 95% CrIs. Each circle represents the pooled estimate, with circle size proportional to the precision of the estimate (inverse of variance), and horizontal lines indicating the corresponding 95% CrIs. Values to the left of the null line indicate a reduction in the outcome, while values to the right indicate an increase.

Abbreviations: XBJ, Xuebijing injection; SF, Shenfu injection; SM, Shenmai injection; SQ, Shenqifuzheng injection; SGM, Shengmai injection; HQ, Huangqi injection; TRQ, Tanreqing injection; WMT, Western medicine treatment.

### 3.5. SUCRA rank

**Fig 5:**
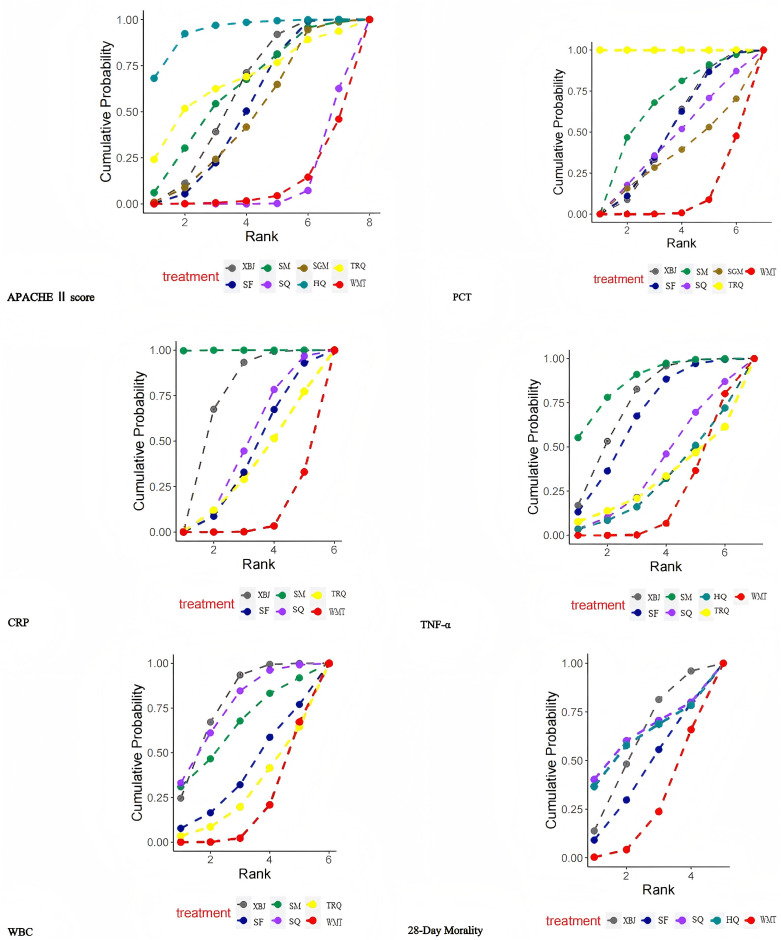
Plots of the Surface Under the Cumulative Ranking Curve (SUCRA) for outcomes.

The detailed rankings and Bayesian characteristics are summarized in Fig 5, while Table 1 visually depicts the probabilistic rankings of different CBDIs when combined with WMT.

**Table 1:** Surface Under the Cumulative Ranking Curve (SUCRA) rank of the outcomes.

Intervention	APACHE Ⅱ score (%)	PCT (%)	CRP (%)	TNF-α (%)	WBC (%)	28-day morality (%)
XBJ + WMT	59.08	49.11	72.04	74.70	76.93	59.84
SF + WMT	51.28	48.90	40.38	67.00	38.40	43.49
SM + WMT	61.99	64.05	99.93	86.85	64.12	–
SQ + WMT	10.01	43.90	46.31	39.64	74.85	62.71
SGM + WMT	47.72	34.45	–	–	–	–
HQ + WMT	93.58	–	–	30.48	–	60.44
TRQ + WMT	66.69	99.99	34.02	30.70	27.59	–
WMT	9.61	9.57	7.30	20.59	18.08	23.49

The SUCRA plots present the cumulative ranking probabilities of each Chinese botanical drug injection combined with WMT across the evaluated outcomes. Higher SUCRA values indicate a greater probability that an intervention ranks among the most effective treatment options for a given outcome.

**AHACHEⅡ score** HQ + WMT achieved the highest probability (93.58%), securing the top rank, followed by TRQ + WMT with a probability of 66.69%. Conversely, WMT alone exhibited the least favorable impact (9.61%).

**PCT** TRQ + WMT achieved the highest probability (99.99%), securing the first rank, followed by SM + WMT (64.05%) in second place. Conversely, WMT alone exhibited the least favorable impact (9.57%).

**CRP** The combination of SM and WMT achieved the highest probability (99.93%), followed by XBJ and WMT (72.04%). Conversely, the standalone use of WMT yielded the least favorable outcome (7.30%).

**TNF-α** SM + WMT achieved the highest probability (86.85%), followed by XBJ + WMT (74.70%). Conversely, WMT alone exhibited the least favorable outcome (20.59%).

**WBC** XBJ + WMT rank first the highest probability (74.70%), following behind is SQ + WMT (74.85%). Conversely, WMT alone exhibited the least favorable outcome (18.08%).

**28-day morality** SQ + WMT has the highest probability (62.71%), securing the top rank, followed by HQ + WMT with a probability of 60.44%. Conversely, WMT alone obtained the least favorable impact (23.49%).

### 3.6. Publication bias

**Fig 6:**
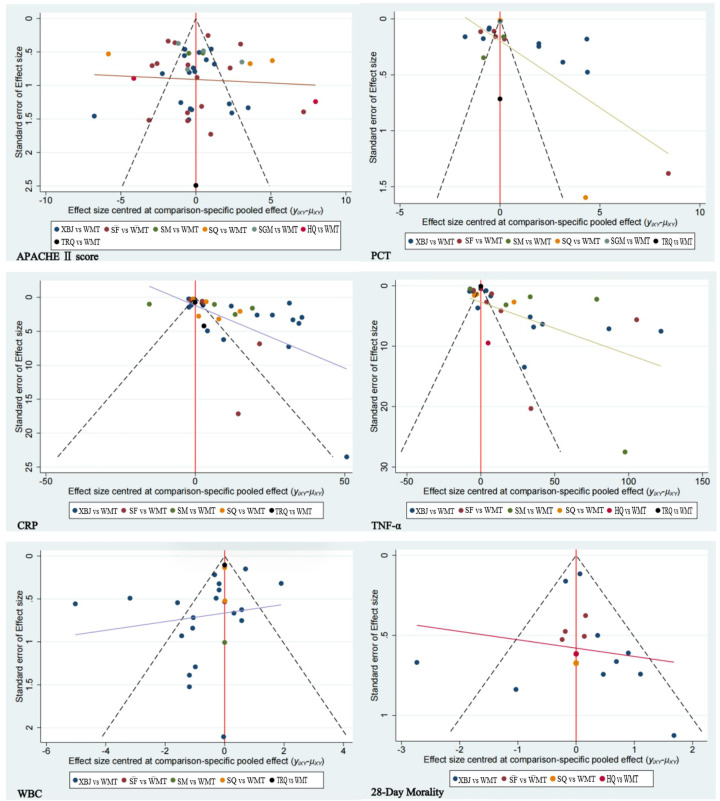
Funnel plot of each outcome indicator.

To evaluate the potential for publication bias within our dataset, comparison-adjusted funnel plots were constructed for each outcome indicator. The symmetry observed in the funnel plots for the APACHE II score suggests an absence of notable publication bias, indicating that the findings for this primary outcome are robust and unbiased. Conversely, the asymmetry detected in funnel plots for other outcome indicators raises concerns about the presence of publication bias. This asymmetry suggests that smaller studies or those with unfavorable outcomes might be underrepresented in the literature, potentially skewing the results of this meta-analysis. These findings are visually represented in Fig 6, providing a graphical interpretation of the distribution and potential biases among the studies included in our analysis.

Each dot represents an individual study included in the network meta-analysis, with the horizontal axis showing the effect size and the vertical axis representing the standard error. Symmetry of the funnel plot suggests a low risk of publication bias, whereas asymmetry may indicate the presence of small-study effects or potential publication bias.

### 3.7. Adverse events

A total of 12 studies reported adverse events, among which 7 studies documented adverse events involving four types of CBDI: XBJ, SF, SQ, and HQ. Five studies reported no occurrence of adverse reactions (SM, SGM). However, only descriptive analyses were performed because the criteria for adverse reactions were not standardized. Detailed results are presented in S6 File.

### 3.8. Heterogeneity analyses and subgroup analyses

The I² statistic, which quantifies the proportion of total variation across studies attributable to heterogeneity rather than chance, was used to assess heterogeneity within each outcome and subgroup. Overall, a high level of variability was observed among the included studies. To explore potential sources of heterogeneity, subgroup analyses were performed by excluding septic shock studies, and by stratifying trials according to patient age and CBDI dosage. When studies involving septic shock were excluded and only sepsis populations were analyzed, the overall effect estimates remained largely consistent with the primary results; however, the number of statistically significant outcomes decreased, suggesting that the presence of septic shock cases may have amplified the treatment effect in the overall analysis. In the age-based subgroup analysis using the APACHE II score as an example, the I² values for patients aged <60 years were 58.1% for XBJ, 93.2% for SF, 39.7% for SM, 99.2% for SQ, and 74.3% for SGM, with an overall heterogeneity of 93.3%. Among patients aged ≥60 years, the corresponding I² values were 75.8% for XBJ, 86.4% for SF, and 98.5% for HQ, resulting in an overall heterogeneity of 89.7%. These results indicate a modest reduction in heterogeneity among the elderly subgroup. In the dose-based subgroup analysis, studies using <100 mL of CBDI reported I² values of 44.52% for XBJ and 98.55% for HQ, with a global I² of 91.21%. For studies administering >100 mL, the I² values were 91.24% for XBJ, 55.99% for SF, and 99.22% for SQ, resulting in an overall heterogeneity of 95.09% (S7 File). These findings suggest that dosage and patient age may partially contribute to the observed heterogeneity across studies.

### 3.9. GRADE quality of evidence

The quality of evidence for the study's outcome indicators was assessed using the GRADE system in the network meta-analysis. This evaluation considered five downgrading factors: publication bias, indirectness, inconsistency, imprecision, and risk of bias. According to GRADE criteria, the evidence for comparisons between interventions ranged from very low to high quality. The primary reasons for downgrading the evidence were risk of bias, followed by inconsistency and imprecision. This indicates that the included studies had deficiencies in experimental design, such as inadequate allocation concealment and lack of blinding. Consequently, the overall quality of evidence was rated as low or moderate (Table 2), and the criterion for judgment of GRADE are presented in S8 File.

**Table 2:** GRADE summary of evidence.

Outcomes	No.	Risk of bias	Inconsistency	Indirectness	Imprecision	Publication bias	GRADE evidence quality
APACHE Ⅱ score	45	High risk of bias	Inconsistency	No indirectness	No imprecision	No publication bias	⊕⊕⊕○○ Moderate quality
PCT	21	High risk of bias	Inconsistency	No indirectness	No imprecision	Publication bias	⊕⊕○○○ Low quality
CRP	34	High risk of bias	Inconsistency	No indirectness	No imprecision	Publication bias	⊕⊕○○○ Low quality
TNF-α	28	High risk of bias	Inconsistency	No indirectness	No imprecision	Publication bias	⊕⊕○○○ Low quality
WBC	26	High risk of bias	Inconsistency	No indirectness	No imprecision	Publication bias	⊕⊕○○○ Low quality
28-day mortality	16	High risk of bias	Inconsistency	No indirectness	No imprecision	No publication bias	⊕⊕⊕○○ Moderate quality

## 4. Discussion

Sepsis, a systemic inflammatory response syndrome triggered by infection, poses a severe threat leading to organ failure and mortality [1]. Despite advancements in medical research, its high morbidity and mortality persist due to complex pathophysiological mechanisms that limit treatment options [91]. Current therapies primarily rely on antibiotics, supportive care, and symptomatic relief, often inadequate in severe sepsis cases [92]. Integrating CBDIs with conventional Western medical treatments is increasingly favored in clinical practice. Our analyses were designed to address key clinical aspects of sepsis management, including identifying CBDI–Western medical treatment (WMT) combinations that most effectively reduce disease severity, alleviate infection-induced inflammation, regulate immune function, and enhance short-term survival.

In this study, we selected APACHE II score as the primary outcome measure, with secondary outcomes including PCT, CRP, TNF-α,WBC, and 28-day mortality. These indicators were chosen based on their widespread clinical application and their significant relevance in assessing sepsis [93,94]. The APACHE II score is extensively used in intensive care units to evaluate the severity of disease in critically ill patients and has demonstrated strong predictive capability for hospital mortality in sepsis cases [95]. PCT serves as a biomarker for bacterial infections and is instrumental in the early diagnosis and management of sepsis [96]. CRP, an acute-phase protein, rises in response to systemic inflammation and is commonly utilized to monitor the inflammatory status of sepsis patients [97]. TNF-α is a key pro-inflammatory cytokine, with elevated levels indicating an intensified inflammatory response characteristic of sepsis [98]. WBC count variations reflect the body's immune response to infection and are critical in evaluating the extent of infection in sepsis patients [99]. Lastly, 28-day mortality is a standard endpoint in sepsis research, providing insight into short-term patient outcomes and treatment efficacy [100]. Collectively, these indicators facilitate a comprehensive assessment of disease severity, inflammatory response, and prognosis in sepsis patients.

### 4.1. Summary of findings

This network meta-analysis (NMA) reviewed 72 RCTs involving 6,351 participants to evaluate the efficacy of seven CBDIs for treating sepsis. It identified five CBDIs (XBJ, SF, SM, SGM, and HQ) that significantly improved APACHE II scores compared to WMT alone. HQ showed the highest likelihood of superior performance in enhancing APACHE II scores. For secondary outcomes, TRQ combined with WMT was most effective in reducing PCT levels, while SM with WMT emerged as the best for lowering both CRP and TNF-α levels. XBJ combined with WMT was optimal for reducing WBC counts, and SQ with WMT showed the greatest effect on improving 28-day mortality rates, although the result was not statistically significant.

The APACHE II score, essential for assessing sepsis severity, integrates various laboratory parameters to reflect tissue damage and assess the body's response to trauma, offering crucial prognostic insights into sepsis outcomes [93]. HQ, among the CBDIs analyzed, significantly improved APACHE II scores, highlighting its potency. Although XBJ combined with WMT showed consistent improvements across several parameters, other interventions such as HQ + WMT and SM + WMT appeared to achieve greater reductions in the more robust APACHE II score, despite being supported by fewer studies. Considering that all included outcomes were graded as having moderate-to-low evidence quality with potential risk of bias and inconsistency (Table 2), these findings should be interpreted cautiously. Further large-scale, rigorously designed RCTs are warranted to validate these observations and to clarify comparative effectiveness across different CBDIs.

Among the secondary outcomes, biomarkers such as PCT, CRP, TNF-α, and WBC serve as critical indicators for assessing the therapeutic response and systemic inflammation in sepsis [1]. In healthy individuals, PCT and CRP levels are typically low (PCT < 0.05 ng/mL; CRP < 10 mg/L), whereas patients with sepsis often exhibit markedly elevated concentrations (PCT > 2 ng/mL; CRP > 100 mg/L), reflecting severe infection and inflammatory activation [5]. Similarly, TNF-α and WBC counts rise sharply during sepsis progression [102]. Therefore, reductions in these biomarkers observed in our network meta-analysis imply attenuation of infection-induced inflammatory burden, providing an objective biochemical basis for evaluating the efficacy of CBDIs combined with WMT.

### 4.2. Implications for clinical practice

CBDIs has shown efficacy in slowing disease progression, reducing mortality rates, shortening antibiotic treatment durations, and lessening adverse events [8]. In TCM theory, sepsis is categorized under infectious diseases characterized by toxicity, stasis, and deficiency. Modern pharmacological studies have demonstrated that TCM properties, such as clearing heat and detoxification, and promoting blood circulation, possess inhibitory effects on viruses.

The SUCRA-based probabilistic rankings provide clinically actionable hierarchies for CBDI selection, revealing distinct therapeutic niches across outcome domains. HQ + WMT demonstrated supremacy in APACHE II (SUCRA 93.58%), with its effect magnitude (MD = −7.96, 95 CI%:-12.21, −3.69) exceeding conventional immunomodulators, positioning it as therapy for high-risk sepsis. TRQ+WMT's dominance in PCT control (SUCRA 99.99%; MD = −20.89, 95%CI = −26.62, −15.17), though its limited CRP efficacy (SUCRA 34.02% vs SM+WMT's 99.93%) necessitates combination strategies for hyperinflammatory phenotypes. SM + WMT emerged as a dual-pathway modulator, simultaneously leading CRP (SUCRA 99.93%) and TNF-α (SUCRA 86.85%) reductions – an effect profile paralleling interleukin-1 antagonists, but with superior safet. Notably, the modest 28-day mortality reduction associated with CBDIs (SQ + WMT SUCRA 62.71%, HQ + WMT 60.44% vs WMT 23.49%) contrasts with their robust biomarker improvements, suggesting primarily adjunctive roles in symptom management rather than definitive mortality mitigation. These findings suggest that specific CBDIs, when combined with WMT, may offer targeted benefits in sepsis management, potentially guiding personalized therapeutic strategies.

In this study, the performance of SF and SGM was generally unremarkable, with neither showing a clear advantage in any specific outcomes. SF primarily contains ginseng and aconite, is traditionally used to warm yang and restore vital energy [101]. However, in sepsis, patients often experience systemic inflammatory response syndrome (SIRS), where excessive immune activation can exacerbate inflammation. SF's yang-warming and qi-tonifying properties may not be well-suited to this context [102]. Comprising ginseng, ophiopogon, and schisandra, SGM is traditionally used to replenish qi, nourish yin, and stabilize the heart [103]. While it can improve cardiac function and reduce fatigue, sepsis involves complex pathophysiology, including multi-organ dysfunction and severe immune dysregulation. The effects of SGM may be insufficient to address these severe and acute conditions.

In our network meta-analysis, we observed a predominance of RCTs focusing on XBJ. This imbalance reflects the current research landscape, where XBJ has been extensively studied for its therapeutic potential in sepsis management. To ensure a comprehensive and unbiased synthesis of available evidence, we included all relevant studies despite this skewness. Incorporating a broader range of interventions and their comparative efficacies allows for a more holistic understanding of treatment options. Moreover, network meta-analytic techniques can accommodate such imbalances by borrowing strength from indirect comparisons, thereby enhancing the robustness of our findings [104]. This approach aligns with established guidelines for conducting network meta-analyses, which recommend including all pertinent evidence to improve the precision and applicability of results.

Many studies have shown that, overall, combination therapy may be more effective than monotherapy. Therefore, early intervention and integration of CBDI and WMT are important tools for improving the cure rate of sepsis and alleviating clinical symptoms.

### 4.3. Safety and subgroup insights

In this study, we observed a significant deficiency in the reporting of AEs among the included RCTs, with only 16.7% of studies providing descriptions, which lacked standardized terminology and classification. Moreover, few studies evaluated the causal relationship between AEs and CBDIs. To enhance reporting quality, future research should adhere to internationally recognized standards, such as the Common Terminology Criteria for Adverse Events (CTCAE) developed by the U.S. National Cancer Institute [105]. Additionally, assessing the causality between AEs and CBDIs is crucial for comprehensively understanding the safety of these treatments. However, due to the complex composition of CBDIs and individual variability in responses, determining causality is challenging. For instance, certain adverse reactions may stem from patient allergies or concomitant medications rather than the CBDIs themselves [106]. We analyzed the safety profiles of each CBDI as outlined in S2 File. These findings are crucial for guiding clinical practice by providing insights into potential side effects. Understanding these safety profiles is essential for tailoring treatment strategies and minimizing risks, particularly in patients with allergies. Further research is warranted, especially regarding long-term safety with extended use [107].

To address these limitations, the certainty of evidence regarding safety outcomes was evaluated using the GRADE framework (S8 File). Specifically, GRADE was applied to assess the overall confidence in safety-related evidence for each CBDI by considering study limitations, inconsistency, indirectness, imprecision, and potential publication bias. Given the sparse and heterogeneous reporting of AEs, most safety outcomes were downgraded due to serious risk of bias and imprecision, indicating that the current evidence supporting the safety profiles of CBDIs is generally of low to very low certainty. Therefore, the GRADE summary was used not to draw definitive comparative safety conclusions, but rather to contextualize the reliability and robustness of the available safety evidence for each intervention.

These findings provide clinically relevant information by identifying potential adverse reactions while simultaneously highlighting the uncertainty of the underlying evidence. This approach supports cautious clinical interpretation and emphasizes the importance of individualized risk assessment, particularly in patients with allergies or multiple comorbidities. Further research is warranted, especially regarding long-term safety with extended use [107]. Addressing these knowledge gaps through additional well-designed clinical trials and observational studies is essential for establishing more robust and evidence-based safety recommendations for CBDIs in the treatment of sepsis.

### 4.4. Strengths and limitations

The main strengths of this meta-analysis are as follows. This study represents a novel attempt to compare seven different CBDIs through a NMA and analyze seven key indicators critical for sepsis treatment, providing the most detailed reference for sepsis management to date. The NMA effectively compares interventions in the absence of direct comparisons, conducting a comprehensive data search across eight databases in both Chinese and English to ensure broad study inclusion. This NMA constitutes the first comprehensive review of a wide range of CBDIs and RCTs, accurately delineating the clinical efficacy of CBDIs in sepsis. However, this study still has several limitations. First, among the 72 included RCTs, there is a high risk of bias, and some outcome measures (e.g., APACHE II) received low GRADE ratings. This has led to certain implementation bias and detection bias for some subjective outcome indicators, such as APACHE Ⅱ. Second, for some CBDIs, such as TRQ and SGM, there were few randomized controlled trials with direct comparison (2 and 4, respectively), and all CBDIs had no direct comparison trials, resulting in increased sensitivity to unbalanced network bias. Third, although SUCRA provided rankings, the clinical applicability of our study results is limited due to the fact that only a small proportion (26.3%) of the included original studies reported the cause of infection, and none addressed patient immune function or treatment costs. In light of these limitations, future research should prioritize large-scale, multicenter, head-to-head, double-blind RCTs to further strengthen the existing evidence and provide more authoritative and clinically relevant guidance for the comprehensive management of sepsis.

## 5. Conclusion

In summary, integrating CBDIs with WMT shows promise in enhancing key sepsis outcomes. Specifically, HQ excels in improving APACHE II scores; TRQ is superior in reducing PCT levels; SM effectively decreases CRP and TNF-α levels; XBJ is noteworthy for lowering WBC counts; and SQ shows a trend toward reducing 28-day mortality rates, although this result was not statistically significant. Notably, XBJ demonstrated consistent advantages across multiple indicators, but further high-quality, large-scale RCTs are required to confirm these findings.

## Supporting information

S1 File PRISMA checklist. Completed PRISMA checklist indicating the location of each reporting item in the manuscript.
(DOCX)

S2 File Details of CBDI included in the trials. Source, metabolite, quality control measure, side effects, interactions, and general safety of each CBDI.
(DOCX)

S3 File The search strategy. The search strategies and results of eight Chinese and English search databases.
(PDF)

S4 File Characteristics of the studies included. Study name, total sample size, age, intervention arm, duration, and outcomes of all included trials.
(DOCX)

S5 File Criterion for judgment of RoB.2. The table presents the risk of bias assessment for included studies, based on the Cochrane ROB 2.0 tool. It evaluates six domains of potential bias—randomization process, deviations from intended interventions, missing outcome data, measurement of the outcome, selection of the reported result, and overall bias—providing an overall judgment of each study’s methodological quality.
(DOCX)

S6 File Occureence of adverse events. Intervention, study, and numbers of adverse events associated with each CBDI across included studies.
(DOCX)

S7 File Heterogeneity and subgroup analyses. Statistical heterogeneity indices (I²), subgroup analysis results based on age, dosage, and disease severity, and corresponding sensitivity analyses.
(DOCX)

S8 File Criterion for judgment of GRADE. Judgment criteria and downgrading rationale applied for assessing the certainty of evidence using the GRADE framework.
(DOCX)
