# Supplementary material for: Comparative efficacy and safety of Chinese botanical drug injection in patients with sepsis: A systematic review and Bayesian network meta-analysis of randomized clinical trials
Source: PLoS One. 2026 Mar 24;21(3):e0343026. doi: 10.1371/journal.pone.0343026 (PMC13012499; doi:10.1371/journal.pone.0343026)
Supplement: S2 File — Source, metabolite, quality control measure, side effects, interactions, and general safety of each CBDI. (DOCX) [file pone.0343026.s002.docx]

Table 1 Details of CBDI included in the trials.

| **Study ID** | **Name** | **Source** | **Metabolite** | **Quality control measure** |
| --- | --- | --- | --- | --- |
| 22-30,  32,36,  41-53,  46-56 | Xuebijing injection | Tianjin Chase Sun Pharmaceutical Co, Ltd.  Z20040033 | Carthamus tinctorius [Asteraceae; Safflower];  Paeonia [Paeoniaceae; Paeonia lactiflora];  Chuanxiong Rhizoma [Apiaceae; Ligusticum chuanxiong];  Salvia miltiorrhiza Bunge [Lamiaceae; Salviae miltiorrhizae radix et rhizoma]  Angelica sinensis (Oliv.) Diels [Apiaceae; Angelica sinensis] | NS |
| 57,59,  61-63,  65-68,  70-73 | Shenfu Injection | China Resources Sanjiu Medical & Pharmaceutical Co., Ltd.  Z51020664 | Panax ginseng[Araliaceae; Ginseng ]  Aconitum carmichaelii Debeaux[Ranunculaceae； Aconitum] | NS |
| 76 | Shenmai Injection | Zhengda Qingchunbao (Deqing) Pharmaceutical Co., Ltd.  Z33020020 | Panax ginseng[Araliaceae; Ginseng ]  Ophiopogon japonicus[Asparagaceae; Ophiopogonis Radix] | NS |
| 77,78 | Shenmai Injection | Shineway Pharmaceutical Co.Ltd  Z13020889 | Panax ginseng[Araliaceae; Ginseng ]  Ophiopogon japonicus[Asparagaceae; Ophiopogonis Radix] | NS |
| 79-83 | Shenqifuzheng Injection | Livzon Pharmaceutical Group Inc.  Z19990065 | Codonopsis pilosula[Campanulaceae; Codonopsis Radix]  Astragalus trimestris[Fabaceae; astragalus membranaceus] | NS |
| 85,86 | Shengmai Injection | China Resources Sanjiu Medical & Pharmaceutical Co., Ltd.  Z51021882 | Panax ginseng[Araliaceae; Ginseng ]  Ophiopogon japonicus[Asparagaceae; Ophiopogonis Radix]  Schisandra chinensis[Schisandraceae; Schisandrae Chinensis Fructus] | NS |
| 87 | Shengmai Injection | Jiangsu Suzhong Pharmaceutical Group Biopharmaceutical Co., Ltd  Z32021056 | Panax ginseng[Araliaceae; Ginseng ]  Ophiopogon japonicus[Asparagaceae; Ophiopogonis Radix]  Schisandra chinensis[Schisandraceae; Schisandrae Chinensis Fructus] | NS |
| 88 | Huangqi Injection | Chengdu Di\Ao Pharmaceutical Group Co., Ltd.  Z51021776 | Astragalus trimestris[Fabaceae; astragalus membranaceus] | NS |
| 89 | Huangqi Injection | Jiangsu Hengfeng Pharmaceutical Co., Ltd.  Z32021257 | Astragalus trimestris[Fabaceae; astragalus membranaceus] | NS |
| 90 | Tanreqing Injection | Shanghai Kaibao Pharmaceutical Co.,Ltd.  Z20030054 | Scutellaria baicalensis [Lamiaceae; Scutellariae Radix]  Xiong dan fen; Goral Horn;  Lonicera[Caprifoliaceae; Lonicerae Japonicae Flos]  Forsythia suspensa[Oleaceae; Forsythiae Fructus] | NS |

NS: not specified.

Table 2. Side effects, interactions, and general safety of CBDI included in the trials.

| **Name** | **Side effects** | **Interactions** | **General safety** |
| --- | --- | --- | --- |
| Xuebijing injection | Occasionally allergic reaction. | NS | 1.Should not be used by pregnant women.  2.Use with caution if allergic to this product. |
| Shenfu  Injection | Occasionally allergic reaction. | NS | Should not be used in people with a history of allergy or serious adverse reactions. |
| Shenmai Injection | Occasionally allergic reaction. | This product should not be mixed with antibiotics  This product contains panax ginseng, should not be used with drugs containing Veratrum nigrum. | Should not be used in newborns, infants. |
| Shenqifuzheng Injection | 1. Mild bleeding may occur in patients with non-Qi-deficiency syndrome after medication.  2. A few patients may have low fever, stomatitis, and lethargy after medication. | This product should not be mixed with chemotherapy drugs. | Should not be used with internal heat, so as not to help heat move blood. |
| Shengmai Injection | 1. Allergic rash.  2. Severe pain in the lower back.  3. Bloating | NS | 1.Should not be used by those allergic to this product.  2.Use with caution to patients with diabetic patients. |
| Huangqi Injection | 1. Allergic reactions.  2. Respiratory system. 3. Circulatory system  4. Digestive system.  5. Others: pyrogen reaction caused by intravenous drip of this product. | NS | Should not be used in patients with a history of allergy or serious adverse reactions. |
| Tanreqing Injection | Occasionally allergic reaction | Should not mix with injections containing acidic ingredients. | Should not be used to the elderly with liver and kidney dysfunction |

NS: not specified.
