# Supplementary material for: Comparative efficacy and safety of Chinese botanical drug injection in patients with sepsis: A systematic review and Bayesian network meta-analysis of randomized clinical trials
Source: PLoS One. 2026 Mar 24;21(3):e0343026. doi: 10.1371/journal.pone.0343026 (PMC13012499; doi:10.1371/journal.pone.0343026)
Supplement: S3 File — The search strategies and results of eight Chinese and English search databases. (PDF) [file pone.0343026.s003.pdf]

1.PubMed：

Search	Query	Items found
#1	(("Sepsis"[Mesh]) OR "Shock, Septic"[Mesh])	144,095
#2	(((((((Sepsis*[Title/Abstract]) OR (Septicemia*[Title/Abstract])) OR (Septic shock*[Title/Abstract])) OR (infectious shock [Title/Abstract])) OR (Systemic Inflammatory Response Syndrome[Title/Abstract])) OR (SIRS[Title/Abstract])) OR (septic[Title/Abstract])) OR (septicaemic shock[Title/Abstract])	184,979
#3	#1 OR #2	258,306
#4	"Injection"[Mesh]	298,172
#5	((Injection[Title/Abstract]) OR (Injectable[Title/Abstract])) OR (Extract[Title/Abstract])	838,512
#6	#4 OR #5	1,030,653
#7	"Randomized Controlled Trial" [Publication Type]	609,105
#8	(((randomized controlled trial[Title/Abstract]) OR (randomized trial[Title/Abstract])) OR (clinic study[Title/Abstract])) OR (clinic trial[Title/Abstract])	166,534
#9	#8 OR #9	667,802
#10	#3 AND #6 AND #9	454

2.Embase：

Search	Query	Items found
#1	'sepsis'/exp	355,348
#2	'septic shock'/exp	74,777
#3	sepsis*:ti OR septicemia*:ti OR 'septic shock*':ti OR ' infectious shock':ti OR 'systemic inflammatory response syndrome':ti OR sirs:ti OR septic:ti OR 'septicaemic shock':ti	93,605
#4	#1 OR #2 OR #3	369,640
#5	'injection'/exp	180,005
#6	'injection':ti OR 'injectable':ti OR 'extract':ti	203,470
#7	#5 OR #6	361,552
#8	'randomized controlled trial'/exp	811,985
#9	'randomized controlled trial ':ti OR 'randomized trial ':ti OR ' clinic study':ti OR 'clinic trial':ti	110,659
#10	#8 OR #9	829,055
#11	#4 AND #7 AND #10	909

3. Cochrane Library：

Search	Query	Items found
#1	MeSH descriptor: [Sepsis] explode all trees	6,463
#2	MeSH descriptor: [Shock, Septic] explode all trees	1,401
#3	(Sepsis OR Septicemia* OR Septic shock* OR infectious shock OR Systemic Inflammatory Response Syndrome OR SIRS OR septic OR septicaemic shock):ti,ab,kw	19,655
#4	#1 OR #2 OR #3	21,653
#5	MeSH descriptor: [Injections] explode all trees	28,033
#6	(injection OR injectable OR extract):ti,ab,kw	177,802
#7	#5 OR #6	177,864
#8	MeSH descriptor: [Randomized Controlled Trial] explode all trees	37
#9	(randomized controlled trial OR randomized trial OR clinic study OR clinic trial):ti,ab,kw	1,267,218
#10	#8 OR #9	1,267,218
#11	#4 AND #7 AND #10	1,319

4. Web of Science core collection：

Search	Query	Items found
#1	TS=(Sepsis OR Septicemia* OR Septic shock* OR infectious shock OR Systemic Inflammatory Response Syndrome OR SIRS OR septic OR septicaemic shock)	246,673
#2	TS=(injection OR injectable OR extract)	2,367,439
#3	TS=(randomized controlled trial OR randomized trial OR clinic study OR clinic trial)	1,006,442
#4	#1 AND #2 AND #3	792

5.中国知网：

Search	Query	Items found
SU%=(脓毒症+脓毒性休克+感染性休克+败血症) AND SU%=(注射液+注射剂+提取物+注射用)		1888

6.万方：

Search	Query	Items found
题名或关键词:(脓毒症 or 脓毒性休克 or 感染性休克 or 败血症) and 题名或关键词:(注射液 or 注射剂 or 提取物 or 注射用)		1383

7.维普：

Search	Query	Items found
(M=脓毒症 OR M=脓毒性休克 OR M=感染性休克 OR M=败血症) AND (M=注射液 OR M=注射剂 OR M=提取物 OR M=注射用)		2151

8.中国生物医学文献数据库：

Search	Query	Items found
("脓毒症"[标题:智能] OR "脓毒性休克"[标题:智能] OR "感染性休克"[标题:智能] OR "败血症"[标题:智能]) AND("注射液"[标题:智能] OR "注射剂"[标题:智能] OR "注射用"[标题:智能] OR "提取物"[标题:智能])		1048
