# Supplementary material for: Comparative efficacy and safety of Chinese botanical drug injection in patients with sepsis: A systematic review and Bayesian network meta-analysis of randomized clinical trials
Source: PLoS One. 2026 Mar 24;21(3):e0343026. doi: 10.1371/journal.pone.0343026 (PMC13012499; doi:10.1371/journal.pone.0343026)
Supplement: S4 File — Study name, total sample size, age, intervention arm, duration, and outcomes of all included trials. (DOCX) [file pone.0343026.s004.docx]

**Table 1 Characteristics of the studies included.**

| **Study** | **Total sample sizes** | **Sample sizes(Sex)** | | **Age(mean±SD)** | | **Intervention arm** | | **Duration**  **(days)** | **Outcome** |
| --- | --- | --- | --- | --- | --- | --- | --- | --- | --- |
|  |  | **T(M/F)** | **C(M/F)** | **T** | **C** | **T** | **C** |  |  |
| Xianquan Liang 2005^[20]^ | 84 | 42 | 42 | — | — | XBJ 50ml bid +WMT | WMT | 14 | ③④ |
| Qihong Chen 2007^[21]^ | 48 | 24  （15/9） | 24  （16/8） | — | — | XBJ 70ml bid +WMT | WMT | 7 | ⑤⑥ |
| Qingquan Liu 2007^[22]^ | 309 | 185（99/86） | 124（64/60） | 45.34±  14.96 | 46.65±  16.44 | XBJ 50ml bid +WMT | WMT | 7 | ⑤ |
| Xun Cai 2008^[23]^ | 154 | 79（40/39） | 75（39/36） | — | — | XBJ 100ml bid +WMT+  Cefooerazone Sodium/  Sulbactam  Sodium 3g bid | WMT+  Cefooerazone Sodium/  Sulbactam Sodium 3g bid | 7 | ⑤ |
| Xuefeng Liu 2008^[24]^ | 120 | 60（37/23） | 60（29/31） | 42.3±  11.3 | 41.7±  14.1 | XBJ 50ml bid +WMT | WMT | 7 | ⑤⑥ |
| Qingbiao Li 2009^[25]^ | 56 | 28  （19/9） | 28  （20/8） | 77±13 | 75±11 | XBJ 100ml q12h +WMT | WMT | 7 | ①④⑥ |
| Yushu Hua 2009^[26]^ | 56 | 31 | 24 | — | — | XBJ 100ml q12h +WMT | WMT | 7 | ①②③⑤ |
| Jianliang Zhu 2010^[27]^ | 38 | 19  （12/7） | 19  （13/6） | 62.1±  13.2 | 65.3±  12.1 | XBJ 50ml bid +WMT+  Mechanical Ventilation | WMT+  Mechanical Ventilation | 7 | ② |
| Xiaojuan Zhang 2010^[28]^ | 32 | 16  （9/7） | 16  （11/5） | 65.25±  15.33 | 64.81±  16.85 | XBJ 100ml bid +WMT | WMT | 5 | ⑥ |
| Da Chen 2011^[29]^ | 80 | 40（21/19） | 40（20/20） | 45.9±  13.9 | 44.8±  14.1 | XBJ 50ml bid +WMT | WMT | 7 | ③④⑤⑥ |
| Lipeng Chen 2011^[30]^ | 60 | 31（17/14） | 29（16/13） | — | — | XBJ 50ml q12h +WMT | WMT | 10 | ①③④⑤ |
| Baocan Jin 2012^[31]^ | 41 | 21 | 20 | — | — | XBJ 100ml bid +WMT | WMT | 7 | ④ |
| Hui Liu 2012^[32]^ | 64 | 32（18/14） | 32（17/15） | 56.3±  10.6 | 55.8±  9.7 | XBJ 50ml bid +WMT | WMT | 8 | ①②③④⑤ |
| Weisheng Liu 2012^[33]^ | 51 | 26（13/13） | 25（13/12） | 70±8 | 74±6 | XBJ 50ml bid +WMT | WMT | 7 | ①⑥ |
| Ronghui Wang 2012^[34]^ | 46 | 23 | 23 | — | — | XBJ 100ml bid +WMT | WMT | 7 | ① |
| Yanping Wang 2012^[35]^ | 60 | 30（17/13） | 30（19/11） | 51.7±  10.2 | 49.3±  14.2 | XBJ 50ml qd +WMT | WMT | 7 | ②⑤ |
| Tingxu Yang 2012^[36]^ | 65 | 33（20/13） | 32（21/11） | 60.15±  14.93 | 61.08±  16.01 | XBJ 50ml bid +WMT | WMT | 6 | ① |
| Yu Wang 2013^[37]^ | 60 | 30 | 30 | — | — | XBJ 50ml bid +WMT | WMT | 7 | ①③④⑤ |
| Yunxia Chen 2013^[38]^ | 731 | 392  （284/  108） | 332（244/88） | — | — | XBJ 100ml bid +WMT | WMT | Untill the symptoms improve | ⑤ |
| Hongli Shen 2013^[39]^ | 40 | 20  （14/6） | 20  （15/5） | 47.25±  13.24 | 51.6±  22.47 | XBJ 100ml bid +WMT | WMT | 7 | ②④ |
| Lina Zhang 2013^[40]^ | 32 | 16  （9/7） | 16  （11/5） | 63.5±  15.23 | 64.58±  17.70 | XBJ 50ml bid +WMT | WMT | 7 | ⑥ |
| Yuancai Liang 2014^[41]^ | 78 | 39（21/18） | 39（22/17） | 62.23±  1.25 | 62.24±  1.24 | XBJ 50ml q12h +WMT | WMT | 7 | ①②③⑤ |
| Lingchun Zhang 2014^[42]^ | 90 | 45 | 45 | 56.9 ± 13.5 | 58.2 ± 12.7 | XBJ 50ml bid +WMT | WMT | 7 | ①②③⑤ |
| Ruiyao Zhu 2014^[43]^ | 66 | 33 | 33 | — | — | XBJ 100ml bid +WMT | WMT | 7 | ①②③⑤ |
| Ningling Dong 2015^[44]^ | 43 | 22 | 21 | — | — | XBJ 50ml q12h +WMT | WMT | 7 | ②③⑤ |
| Yakuan Wang 2015^[45]^ | 40 | 20  （12/8） | 20  （11/9） | 67.1±  13.5 | 68.9±  12.4 | XBJ 50ml bid +WMT | WMT | 7 | ①⑥ |
| Minggang Yu 2016^[46]^ | 68 | 34（21/13） | 34（19/15） | 33.15±  25.1 | 33.85±  25.4 | XBJ 100ml bid +WMT | WMT | 3—7 | ①⑤ |
| Chengjuan Du 2016^[47]^ | 60 | 30（14/16） | 30（15/15） | 48.5±  1.3 | 47.8±  1.5 | XBJ 100ml bid +WMT | WMT | 7 | ① |
| Duanhui Liu 2016^[48]^ | 120 | 60（35/25） | 60（32/28） | 50±6 | 49±5 | XBJ 50ml bid +WMT | WMT | 14 | ②③④⑤ |
| Si Chen 2018^[49]^ | 78 | 39（21/18） | 39（23/16） | 47.32 ±5.2 | 47.24 ±5.31 | XBJ 50ml q12h +WMT+  Levosimendan 0.2ug q24h | WMT+ Levosimendan 0.2ug q24h | 7 | ① |
| Jingxing Zhong 2019^[50]^ | 76 | 38（20/18） | 38（21/17） | 58. 98 ±9.22 | 58. 75 ±  9. 14 | XBJ 50ml qd +WMT+Blood Purification 2000ml/h | WMT+ Blood Purification 2000ml/h | 7 | ①③④ |
| Nan Li 2020^[51]^ | 66 | 33（18/15） | 33（17/16） | 63.42±  1.42 | 63.36±  1.4 | XBJ 100ml bid +WMT+Blood Purification 2000-3000ml/h 24h | WMT+Blood Purification 2000-3000ml/h 24h | 5 | ②③⑤ |
| Yunhai Zhang 2021^[52]^ | 86 | 44（25/19） | 42（24/18） | 65.99±  12.23 | 63.24±  11.86 | XBJ 100ml bid +WMT | WMT | 5 | ①④⑥ |
| Chengxin Hu 2022^[53]^ | 82 | 41（26/15） | 41（24/17） | 58.96±  4.35 | 59.05±  4.39 | XBJ 50ml bid +WMT  +ulinastatin 200KU bid | WMT+ ulinastatin 200KU bid | 7 | ②④ |
| Guanjiao Zhou 2023^[54]^ | 136 | 68（36/32） | 68（38/30） | 58. 13 ±4. 72 | 57. 36 ±  4. 89 | XBJ 50ml bid +WMT+Blood Purification 60ml/（kg·h） 24h | WMT+ Blood Purification 60ml/（kg·h） 24h | 7 | ①③⑤ |
| Yefen Zhou 2023^[55]^ | 82 | 41（27/14） | 41（30/17） | 67.02±  5.73 | 67.32±  5.11 | XBJ 50ml bid +WMT+  linezolid 600mg q12h | WMT+ linezolid 600mg q12h | 10 | ① |
| Songqiao Liu 2023^[56]^ | 1817 | 911  （580/  331） | 906  （619/  287） | 56.3±13.4 | 56.8±13.6 | XBJ 100ml q12h +WMT | WMT | 5 | ⑥ |
| Jianqi Ma 2015^[57]^ | 120 | 60（32/28） | 60（32/28） | 51.3±  4.3 | 49.3±  5.3 | SF +WMT+  ulinastatin 100KU bid | WMT+ ulinastatin 100KU bid | 7 | ①④ |
| Guogang Zhang 2015^[58]^ | 60 | 30（14/16） | 30（18/12） | 50.50±  17.20 | 50.50±  17.25 | SF 100ml qd +WMT | WMT | 14 | ①③ |
| Dejun Wu 2014^[59]^ | 81 | 42（25/17） | 39（23/16） | 49. 2 ±  16. 5 | 48.6 ±  16.2 | SF 100ml qd +WMT | WMT | 14 | ① |
| Yangchun Xiao 2017^[60]^ | 71 | 36（23/13） | 35（22/13） | 65.72±  12.24 | 66.42±  13.75 | SF 100ml qd +WMT | WMT | 5 | ①⑥ |
| Shiyuan Zhang 2017^[61]^ | 71 | 36（19/17） | 35（20/15） | 71.43±  9.21 | 69.37±  10.35 | SF 100ml qd +WMT | WMT | 7 | ①⑥ |
| Tianchang Cheng 2018^[62]^ | 68 | 34（23/11） | 34（21/13） | 56.65±  8.17 | 57.33±  7.29 | SF 100ml qd +WMT | WMT | 7 | ①②④ |
| Xing Li 2019^[63]^ | 64 | 32（18/14） | 32（19/13） | 49.1±  15.7 | 49.2±  15.4 | SF 100ml qd +WMT | WMT | 7 | ① |
| Rong Xu 2019^[64]^ | 68 | 34（20/14） | 34（18/16） | 26.63±  3.78 | 49.12±  9.16 | SF 100ml qd +WMT+Blood Purification 2000ml/h | WMT+ Blood Purification 2000ml/h | 7 | ①②③④⑥ |
| Yanqing Guo 2020^[65]^ | 84 | 42（24/18） | 42（23/49） | 57.24±  6.29 | 56.15±  5.74 | SF 100ml qd +WMT+  hydrocortisone 100mg qd | WMT+ hydrocortisone 100mg qd | 7 | ② |
| Li Yang 2021^[66]^ | 96 | 48（22/26） | 48（23/25） | 46.73±  7.72 | 46.28±  7.81 | SF 60ml qd +WMT+  hydrocortisone 100mg qd | WMT+ hydrocortisone 100mg qd | 14 | ①②④ |
| Hongyan Wang 2021^[67]^ | 100 | 50（27/23） | 50（26/24） | 62.15±  2.58 | 61.82±  2.56 | SF 100ml qd +WMT+  dexamethasone sodium phosphate 2-20mg | WMT+ dexamethasone sodium phosphate 2-20mg | 7 | ①②③ |
| Shuai Xu 2016^[68]^ | 93 | 47（29/18） | 46（27/19） | 52.2±  7.2 | 52.3 ±  7.2 | SF 60ml +WMT+  hydrocortisone 100mg qd | WMT+ hydrocortisone 100mg qd | 5 | ①②③ |
| Qimin Xiong 2022^[69]^ | 60 | 30（17/13） | 30（18/12） | 46.75±  15.20 | 47.00±  15.25 | SF 100ml qd +WMT | WMT | 7 | ②④ |
| Kuiwen Gong 2023^[70]^ | 72 | 36（20/16） | 36（21/15） | 52.43±  5.01 | 53.56±  5.13 | SF 100ml qd +WMT | WMT | 14 | ①④ |
| Run Li 2023^[71]^ | 100 | 50（29/21） | 50（30/20） | 51.86 ±  8.74 | 52.45±  8.93 | SF 100ml qd +WMT+ Blood Purification 1800-2500ml/h 8h/d | WMT+ Blood Purification 1800-2500ml/h 8h/d | 7 | ① |
| Mansheng Zeng 2023^[72]^ | 100 | 50（28/22） | 50（27/23） | 62.36±  7.56 | 62.85±  7.63 | SF 60ml +WMT | WMT | 5 | ① |
| Ning Zhang 2017^[73]^ | 157 | 78（43/35） | 79（45/34） | 59.3±  16.4 | 58.6±  17.2 | SF 100ml qd +WMT | WMT | 7 | ①④⑥ |
| Dao Zeng 2013^[74]^ | 50 | 25 | 25 | — | — | SM 60ml +WMT | WMT | 10 | ②④ |
| Jifeng Bao 2015^[75]^ | 112 | 56 | 56 | — | — | SM 60/100ml +WMT | WMT | 7 | ③④ |
| Xiaoyun Xu 2015^[76]^ | 80 | 40 | 40 | — | — | SM 10ml/h+WMT | WMT | 7 | ②③④⑤ |
| Wenyue Liu 2019^[77]^ | 142 | 71（42/29） | 71（38/33） | 52.48±  11.62 | 51.67±  11.19 | SF 100ml qd +WMT+Levosimendan0.075ug/kg/min 24h | WMT+Levosimendan0.075ug/kg/min 24h | 7 | ①④ |
| Baozhu Shi 2019^[78]^ | 106 | 53（37/16） | 53（35/18） | 50.3±  13.1 | 50.5±  13.2 | SF 200ml qd +WMT | WMT | 5 | ①②③④ |
| Zhirong Huo 2017^[79]^ | 92 | 46（24/22） | 46（25/21） | 51. 73 ±10.14 | 49. 67 ±  9. 21 | SQ 250ml qd +WMT+ Blood Purification 3000-4000mL/min 12-24h | WMT+ Blood Purification 3000-4000mL/min 12-24h | 15 | ①②③④ |
| Ping Li 2018^[80]^ | 62 | 31 | 31 | — | — | SQ 100ml qd +WMT | WMT | 7 | ②⑤ |
| Weimin Hu 2019^[81]^ | 78 | 39（25/14） | 39（24/15） | 55.29±  4.52 | 54.78±  4.63 | SQ 250ml qd +WMT+ ulinastatin 600KU bid | WMT+ ulinastatin 600KU bid | 7 | ①③④⑥ |
| Qin Fang 2022^[82]^ | 90 | 45（29/16） | 45（27/18） | 45.90±  4.17 | 45.21±  4.37 | SQ 250ml qd +WMT+  hydrocortisone 100mg q8h | WMT+ hydrocortisone 100mg q8h | 7 | ①③ |
| Fan Zhang 2023^[83]^ | 100 | 50（27/23） | 50（25/25） | 53.2±7.5 | 53. 5±  7. 2 | SQ 250ml qd +WMT+  moxifloxacin 400mg q8h | WMT+ moxifloxacin 400mg q8h | 14 | ②③④⑤ |
| Yaling Sun 2017^[84]^ | 30 | 15  （9/7） | 15  （10/5） | 76.4 | 73.2 | SGM 60ml qd+WMT+  Norepinephrine 0.3mg/kg | WMT+ Norepinephrine 0.3mg/kg | 3 | ① |
| Taotao He 2021^[85]^ | 84 | 42（25/17） | 42（23/19） | 51.74±  6.08 | 51.39±  6.14 | SGM 60ml qd+WMT+  Ultrasound-Guided fluid resuscitation | WMT+ Ultrasound-Guided fluid resuscitation | 3 | ① |
| Jun Yao 2021^[86]^ | 100 | 50（28/22） | 50（30/20） | 58.8±  3.2 | 59.2±  3.4 | SGM 60ml qd+WMT | WMT | 7 | ①② |
| Chao Liu 2022^[87]^ | 83 | 41（24/17） | 41（25/17） | 50.28±  7.3 | 50.35±  7.66 | SGM 60ml qd+WMT | WMT | 7 | ①② |
| Qun Su 2009^[88]^ | 50 | 30  （20/10） | 20  （15/5） | 73.80±  9.50 | 75.35±  10.18 | HQ 30ml bid+WMT | WMT | 14 | ①④⑥ |
| Minhui Wang 2022^[89]^ | 78 | 39（23/16） | 39（25/14） | 60.72±  6.23 | 59.67±  6.06 | HQ 60ml qd+WMT | WMT | 14 | ①④ |
| Zhi Liu 2011^[90]^ | 66 | 35 | 31 | — | — | TRQ 20ml qd+WMT | WMT | 7 | ①③⑥ |
| Sanjun Zhou 2014^[91]^ | 85 | 47（29/18） | 38（20/18） | — | — | TRQ 30ml qd+WMT | WMT | 7 | ②③④ |

Note: XBJ:Xuebijing injection; SF:Shenfu injection; SM:Shenmai injection; SQ:Shenqifuzheng injection; SGM:Shengmai injection; HQ:Huangqi injection; TRQ:Tanreqing injection. WMT:Western medicine treatments. ①:Acute Physiology and Chronic Health Evaluation (APACHE) II score; ②:procalcitonin(PCT); ③:C-reactive protein(CRP); ④:tumor necrosis factor-α(TNF-α); ⑤:white blood cell count(WBC); ⑥:28-day mortality.

Symbols: “ - ” means “without”
