# Supplementary material for: Comparative efficacy and safety of Chinese botanical drug injection in patients with sepsis: A systematic review and Bayesian network meta-analysis of randomized clinical trials
Source: PLoS One. 2026 Mar 24;21(3):e0343026. doi: 10.1371/journal.pone.0343026 (PMC13012499; doi:10.1371/journal.pone.0343026)
Supplement: S5 File — The table presents the risk of bias assessment for included studies, based on the Cochrane ROB 2.0 tool. It evaluates six domains of potential bias—randomization process, deviations from intended interventions, missing outcome data, measurement of the outcome, selection of the reported result, and overall bias—providing an overall judgment of each study’s methodological quality. (DOCX) [file pone.0343026.s005.docx]

| **Table 1. Criterion for judgment of RoB.2** | | | | | | |
| --- | --- | --- | --- | --- | --- | --- |
|  | **Randomization process** | **Deviations from intended interventions** | **Mising outcome data** | **Measurement of the outcome** | **Selection of the reported result** | **Overall Bias** |
| Xianquan Liang 2005^[16]^ | High risk | Some concerns | Low risk | Low risk | Some concerns | High risk |
| Qihong Chen 2007^[17]^ | High risk | Some concerns | Low risk | Low risk | Some concerns | High risk |
| Qingquan Liu 2007^[18]^ | High risk | Some concerns | Low risk | Low risk | Some concerns | High risk |
| Xun Cai 2008^[19]^ | High risk | Some concerns | Low risk | Low risk | Some concerns | High risk |
| Xuefeng Liu 2008^[20]^ | High risk | Some concerns | Low risk | Low risk | Some concerns | High risk |
| Qingbiao Li 2009^[21]^ | High risk | Some concerns | Low risk | Low risk | Some concerns | High risk |
| Yushu Hua 2009^[22]^ | High risk | Low risk | Low risk | Low risk | Some concerns | High risk |
| Jianliang Zhu 2010^[23]^ | Low risk | Some concerns | Low risk | Low risk | Low risk | Low risk |
| Xiaojuan Zhang 2010^[24]^ | Some concerns | Some concerns | Low risk | Low risk | Some concerns | Some concerns |
| Da Chen 2011^[25]^ | High risk | Some concerns | Low risk | Low risk | Some concerns | High risk |
| Lipeng Chen 2011^[26]^ | High risk | Some concerns | Low risk | Low risk | Some concerns | High risk |
| Baocan Jin 2012^[27]^ | High risk | Some concerns | Low risk | Low risk | Some concerns | High risk |
| Hui Liu 2012^[28]^ | High risk | Some concerns | Low risk | Low risk | Some concerns | High risk |
| Weisheng Liu 2012^[29]^ | High risk | Some concerns | Low risk | Low risk | Some concerns | High risk |
| Ronghui Wang 2012^[30]^ | High risk | Some concerns | Low risk | Low risk | Some concerns | High risk |
| Yanping Wang 2012^[31]^ | High risk | Some concerns | Low risk | Low risk | Some concerns | High risk |
| Tingxu Yang 2012^[32]^ | High risk | Some concerns | Low risk | Low risk | Some concerns | High risk |
| Yu Wang 2013^[33]^ | High risk | Some concerns | Low risk | Low risk | Some concerns | High risk |
| Yunxia Chen 2013^[34]^ | Some concerns | Some concerns | Low risk | Low risk | Some concerns | Some concerns |
| Hongli Shen 2013^[35]^ | High risk | Some concerns | Low risk | Low risk | Some concerns | High risk |
| Lina Zhang 2013^[36]^ | High risk | Some concerns | Low risk | Low risk | Some concerns | High risk |
| Yuancai Liang 2014^[37]^ | High risk | Some concerns | Low risk | Low risk | Some concerns | High risk |
| Lingchun Zhang 2014^[38]^ | Some concerns | Some concerns | Low risk | Low risk | Some concerns | Some concerns |
| Ruiyao Zhu 2014^[39]^ | High risk | Some concerns | Low risk | Low risk | Some concerns | High risk |
| Ningling Dong 2015^[40]^ | High risk | Some concerns | Low risk | Low risk | Some concerns | High risk |
| Yakuan Wang 2015^[41]^ | High risk | Some concerns | Low risk | Low risk | Some concerns | High risk |
| Minggang Yu 2016^[42]^ | High risk | Some concerns | Low risk | Low risk | Some concerns | High risk |
| Chengjuan Du 2016^[43]^ | Some concerns | Some concerns | Low risk | Low risk | Some concerns | Some concerns |
| Duanhui Liu 2016^[44]^ | Some concerns | Some concerns | Low risk | Low risk | Some concerns | Some concerns |
| Si Chen 2018^[45]^ | Some concerns | Some concerns | Low risk | Low risk | Some concerns | Some concerns |
| Jingxing Zhong 2019^[46]^ | Some concerns | Some concerns | Low risk | Low risk | Some concerns | Some concerns |
| Nan Li 2020^[47]^ | Some concerns | Some concerns | Low risk | Low risk | Some concerns | Some concerns |
| Yunhai Zhang 2021^[48]^ | Some concerns | Some concerns | Low risk | Low risk | Some concerns | Some concerns |
| Chengxin Hu 2022^[49]^ | Some concerns | Some concerns | Low risk | Low risk | Some concerns | Some concerns |
| Guanjiao Zhou 2023^[50]^ | High risk | Some concerns | Low risk | Low risk | Some concerns | High risk |
| Yefen Zhou 2023^[51]^ | Some concerns | Low risk | Low risk | Low risk | Some concerns | Some concerns |
| Songqiao Liu 2023^[52]^ | Low risk | Some concerns | Low risk | Low risk | Low risk | Low risk |
| Jianqi Ma 2015^[53]^ | Some concerns | Low risk | Low risk | Low risk | Some concerns | Some concerns |
| Guogang Zhang 2015^[54]^ | Some concerns | Low risk | Low risk | Low risk | Some concerns | Some concerns |
| Dejun Wu 2014^[55]^ | High risk | Low risk | Low risk | Low risk | Some concerns | High risk |
| Yangchun Xiao 2017^[56]^ | Some concerns | Low risk | Low risk | Low risk | Some concerns | Some concerns |
| Shiyuan Zhang 2017^[57]^ | Some concerns | Low risk | Low risk | Low risk | Some concerns | Some concerns |
| Tianchang Cheng 2018^[58]^ | Some concerns | Low risk | Low risk | Low risk | Some concerns | Some concerns |
| Xing Li 2019^[59]^ | Some concerns | Low risk | Low risk | Low risk | Some concerns | Some concerns |
| Rong Xu 2019^[60]^ | Some concerns | Low risk | Low risk | Low risk | Some concerns | Some concerns |
| Yanqing Guo 2020^[61]^ | Some concerns | Low risk | Low risk | Low risk | Some concerns | Some concerns |
| Li Yang 2021^[62]^ | Some concerns | Low risk | Low risk | Low risk | Some concerns | Some concerns |
| Hongyan Wang 2021^[63]^ | Some concerns | Low risk | Low risk | Low risk | Some concerns | Some concerns |
| Shuai Xu 2016^[64]^ | Some concerns | Some concerns | Low risk | Low risk | Some concerns | Some concerns |
| Qimin Xiong 2022^[65]^ | Some concerns | Some concerns | Low risk | Low risk | Some concerns | Some concerns |
| Kuiwen Gong 2023^[66]^ | Some concerns | Some concerns | Low risk | Low risk | Some concerns | Some concerns |
| Run Li 2023^[67]^ | Some concerns | Some concerns | Low risk | Low risk | Some concerns | Some concerns |
| Mansheng Zeng 2023^[68]^ | Some concerns | Some concerns | Low risk | Low risk | Some concerns | Some concerns |
| Ning Zhang 2017^[69]^ | Low risk | Some concerns | Low risk | Low risk | Low risk | Low risk |
| Dao Zeng 2013^[70]^ | High risk | Some concerns | Low risk | Low risk | Some concerns | High risk |
| Jifeng Bao 2015^[71]^ | Some concerns | Some concerns | Low risk | Low risk | Some concerns | Some concerns |
| Xiaoyun Xu 2015^[72]^ | High risk | Some concerns | Low risk | Low risk | Some concerns | High risk |
| Wenyue Liu 2019^[73]^ | Some concerns | Some concerns | Low risk | Low risk | Some concerns | Some concerns |
| Baozhu Shi 2019^[74]^ | Some concerns | Some concerns | Low risk | Low risk | Some concerns | Some concerns |
| Zhirong Huo 2017^[75]^ | Some concerns | Some concerns | Low risk | Low risk | Some concerns | Some concerns |
| Ping Li 2018^[76]^ | Some concerns | Some concerns | Low risk | Low risk | Some concerns | Some concerns |
| Weimin Hu 2019^[77]^ | Some concerns | Some concerns | Low risk | Low risk | Some concerns | Some concerns |
| Qin Fang 2022^[78]^ | Some concerns | Some concerns | Low risk | Low risk | Some concerns | Some concerns |
| Fan Zhang 2023^[79]^ | Some concerns | Some concerns | Low risk | Low risk | Some concerns | Some concerns |
| Yaling Sun 2017^[80]^ | High risk | Some concerns | Low risk | Low risk | Some concerns | High risk |
| Taotao He 2021^[81]^ | Some concerns | Some concerns | Low risk | Low risk | Some concerns | Some concerns |
| Jun Yao 2021^[82]^ | Some concerns | Some concerns | Low risk | Low risk | Some concerns | Some concerns |
| Chao Liu 2022^[83]^ | Some concerns | Some concerns | Low risk | Low risk | Some concerns | Some concerns |
| Qun Su 2009^[84]^ | High risk | Some concerns | Low risk | Low risk | Some concerns | High risk |
| Minhui Wang 2022^[85]^ | Some concerns | Some concerns | Low risk | Low risk | Some concerns | Some concerns |
| Zhi Liu 2011^[86]^ | High risk | Some concerns | Low risk | Low risk | Some concerns | High risk |
| Sanjun Zhou 2014^[87]^ | High risk | Some concerns | Low risk | Low risk | Some concerns | High risk |
