# Supplementary material for: Comparative efficacy and safety of Chinese botanical drug injection in patients with sepsis: A systematic review and Bayesian network meta-analysis of randomized clinical trials
Source: PLoS One. 2026 Mar 24;21(3):e0343026. doi: 10.1371/journal.pone.0343026 (PMC13012499; doi:10.1371/journal.pone.0343026)
Supplement: S6 File — Intervention, study, and numbers of adverse events associated with each CBDI across included studies. (DOCX) [file pone.0343026.s006.docx]

**Table 1. Occurrence of adverse events.**

| **Intervention** | **Study** | **Adverse events** | **T** | **C** |
| --- | --- | --- | --- | --- |
| XBJ+WMT vs. WMT | Qingbiao Li2009^[25]^ | Skin rashes or itching | 1 | 0 |
|  | Yunxia Chen2013^[38]^ | - | - | - |
|  | Chengxin Hu2022^[53]^ | - | - | - |
|  | Yefen Zhou2023^[55]^ | Skin rashes or itching | 1 | 0 |
|  |  | Diarrhoea | 1 | 1 |
|  |  | Nausea | 1 | 1 |
|  |  | Headache | 0 | 1 |
|  | Songqiao Liu2023^[56]^ | Glutamic-oxaloacetic | 36 | 38 |
|  |  | WBC count increased | 38 | 36 |
|  |  | Hemoglobin decreased | 31 | 35 |
|  |  | Platelet count decreased | 27 | 24 |
|  |  | BUN increased | 30 | 18 |
|  |  | Bilirubin total increased | 26 | 16 |
|  |  | APTT prolonged | 19 | 17 |
|  |  | Fibrinogen increased | 17 | 7 |
|  |  | Platelet count increased | 9 | 14 |
|  |  | Urine WBC increased | 8 | 15 |
|  |  | Prothrombin time increased | 13 | 9 |
|  |  | Urine RBC increased | 11 | 8 |
|  |  | Blood creatinine increased | 11 | 6 |
|  |  | Sugar blood increased | 7 | 8 |
|  |  | Blood creatinine decreased | 6 | 7 |
|  |  | Fibrin D dimer increased | 5 | 8 |
|  |  | Urinary protein increased | 6 | 7 |
|  |  | Decreased white cell count | 4 | 6 |
|  |  | Glucose urine elevated | 7 | 3 |
|  |  | Fibrinogen decreased | 6 | 3 |
|  |  | Faecal occult blood positive | 3 | 4 |
|  |  | BUN decreased | 1 | 3 |
|  |  | AST decreasd | 2 | 0 |
|  |  | Blood pressure decreased | 2 | 0 |
|  |  | Blood pressure increased | 0 | 2 |
|  |  | ALT decreased | 1 | 0 |
| SF+WMT vs. WMT | Jianqi Ma2015^[57]^ | - | - | - |
|  | Shuai Xu2016^[68]^ | Gastrointestinal reaction | 1 | 3 |
|  |  | Headache | 1 | 2 |
|  |  | Skin rashes or itching | 2 | 1 |
| SM+WMT vs. WMT | Dao Zeng2013^[74]^ | - | - | - |
| SQ+WMT vs. WMT | Qin Fang2022^[82]^ | Dizziness | 2 | 1 |
|  |  | Fatigue | 1 | 1 |
|  |  | Nausea | 2 | 1 |
|  |  | Muscle pain | 1 | 2 |
|  | Fan Zhang2023^[83]^ | Skin rashes or itching | 3 | 1 |
|  |  | Nausea | 2 | 2 |
|  |  | Headache | 1 | 1 |
| SGM+WMT vs. WMT | Jun Yao2021^[86]^ | - | - | - |
| HQ+WMT vs. WMT | Minhui Wang2022^[89]^ | Diarrhoea | 1 | 2 |
|  |  | Skin rashes or itching | 0 | 1 |
