# Supplementary material for: Comparative efficacy and safety of Chinese botanical drug injection in patients with sepsis: A systematic review and Bayesian network meta-analysis of randomized clinical trials
Source: PLoS One. 2026 Mar 24;21(3):e0343026. doi: 10.1371/journal.pone.0343026 (PMC13012499; doi:10.1371/journal.pone.0343026)
Supplement: S7 File — Statistical heterogeneity indices (I²), subgroup analysis results based on age, dosage, and disease severity, and corresponding sensitivity analyses. (DOCX) [file pone.0343026.s007.docx]

**1 Heterogeneity analyses**

1.1 All studies’ heterogeneity analyses

1.1.1 APACHE Ⅱ

|  | i^2^.pair | i^2^.cons | incons.p |
| --- | --- | --- | --- |
| XBJ | 70.55304 | 70.55277 | NA |
| SF | 92.61495 | 92.61323 | NA |
| SM | 39.06803 | 39.07325 | NA |
| SQ | 99.10299 | 99.10254 | NA |
| SGM | 91.84430 | 91.83242 | NA |
| HQ | 98.52843 | 98.52752 | NA |
| TRQ | NA | NA | NA |
| Global I-squared: | 93.41017 | 93.40729 | NA |

1.1.2 PCT

|  | i^2^.pair | i^2^.cons | incons.p |
| --- | --- | --- | --- |
| XBJ | 99.56612 | 99.56714 | NA |
| SF | 99.87252 | 99.87220 | NA |
| SM | 91.32951 | 91.55759 | NA |
| SQ | 99.99530 | 99.99534 | NA |
| SGM | NA | NA | NA |
| TRQ | NA | NA | NA |
| Global I-squared: | 99.9446 | 99.94488 | NA |

1.1.3 CRP

|  | i^2^.pair | i^2^.cons | incons.p |
| --- | --- | --- | --- |
| XBJ | 99.85619 | 99.85627 | NA |
| SF | 99.12318 | 99.12429 | NA |
| SM | 99.43312 | 99.43249 | NA |
| SQ | 99.39574 | 99.39476 | NA |
| TRQ | 74.21844 | 72.67174 | NA |
| Global I-squared: | 93.41017 | 93.40729 | NA |

1.1.4 TNF-α

|  | i^2^.pair | i^2^.cons | incons.p |
| --- | --- | --- | --- |
| XBJ | 99.79223 | 99.79269 | NA |
| SF | 99.76966 | 99.76887 | NA |
| SM | 99.95457 | 99.95444 | NA |
| SQ | 98.13999 | 98.14027 | NA |
| HQ | 95.83039 | 95.96570 | NA |
| TRQ | NA | NA | NA |
| Global I-squared: | 99.86183 | 99.86162 | NA |

1.1.5 WBC

|  | i^2^.pair | i^2^.cons | incons.p |
| --- | --- | --- | --- |
| XBJ | 93.04784 | 93.03478 | NA |
| SF | NA | NA | NA |
| SM | NA | NA | NA |
| SQ | 0.00000 | 0.00000 | NA |
| TRQ | NA | NA | NA |
| Global I-squared: | 92.66174 | 92.64784 | NA |

1.1.6 28-day mortality

|  | i^2^.pair | i^2^.cons | incons.p |
| --- | --- | --- | --- |
| XBJ | 70.53533 | 70.50642 | NA |
| SF | 0.00000 | 0.00000 | NA |
| SQ | NA | NA | NA |
| HQ | NA | NA | NA |
| Global I-squared: | 61.54619 | 61.50459 | NA |

1.2 Patients aged <60 years

| Years | CBDI | Heterogeneity（I^2^ (95% CI)） | Heterogeneity  assessment |
| --- | --- | --- | --- |
| ＜60 | XBJ | 58.07577 | Substantial |
|  | SF | 93.19351 | High |
|  | SM | 39.66817 | Moderate |
|  | SQFZ | 99.21344 | High |
|  | SHENGM | 74.33947 | Substantial |
|  | Global | 93.2892 | High |
| ＞60 | XBJ | 75.79969 | High |
|  | SF | 86.43244 | High |
|  | HQ | 98.53453 | High |
|  | TRQ | NA | NA |
|  | Global | 89.73745 | High |

1.3 The dose about 100 ml

| Dose of drug administered | CBDI | Heterogeneity（I^2^ (95% CI)） | Heterogeneity  assessment |
| --- | --- | --- | --- |
| ＜100ml | XBJ | 44.51908 | Moderate |
|  | HQ | 98.54768 | High |
|  | TRQ | NA | NA |
|  | Global | 91.21377 | High |
| ＞100ml | XBJ | 91.24400 | High |
|  | SF | 55.99240 | Substantial |
|  | SQ | 99.22205 | High |
|  | Global | 95.09041 | High |

**2 Subgroup analyses**

2.1 Excluding the septic shock studies

Table1 League tables comparing interventions of APACHE Ⅱ score.

| **XBJ+**  **WMT** |  |  |  |  |  |  |
| --- | --- | --- | --- | --- | --- | --- |
| -1.28 (-4.5, 1.96) | **SF+**  **WMT** |  |  |  |  |  |
| 0.91 (-6.02, 7.97) | 2.21 (-5, 9.4) | **SM+**  **WMT** |  |  |  |  |
| -4 (-8.28, 0.34) | -2.73 (-7.38, 1.95) | -4.91 (-12.69, 2.84) | **SQ+**  **WMT** |  |  |  |
| 4.3 (-0.92, 9.63) | 5.58 (-0.01, 11.21) | 3.39 (-4.93, 11.77) | **8.3 (2.08, 14.59)** | **HQ+**  **WMT** |  |  |
| 1.44 (-7.03, 9.84) | 2.71 (-6.01, 11.3) | 0.52 (-10.14, 11.09) | 5.44 (-3.73, 14.59) | -2.85 (-12.5, 6.7) | **TRQ+**  **WMT** |  |
| **-3.71 (-5.57, -1.84)** | -2.43 (-5.03, 0.17) | -4.63 (-11.38, 2.06) | 0.29 (-3.61, 4.19) | **-8.01 (-13, -3.1)** | -5.14 (-13.33, 3.17) | **WMT** |

Table2 League tables comparing interventions of PCT

| **XBJ+**  **WMT** |  |  |  |  |  |
| --- | --- | --- | --- | --- | --- |
| 0.69 (-3.17, 4.74) | **SF+**  **WMT** |  |  |  |  |
| 0.72 (-6.16, 7.61) | 0.03 (-7.36, 7.26) | **SM+**  **WMT** |  |  |  |
| -0.16 (-5.36, 5.24) | -0.84 (-6.74, 4.96) | -0.9 (-8.88, 7.33) | **SQ+**  **WMT** |  |  |
| **18.72 (11.71, 25.7)** | **18.03 (10.52, 25.36)** | **18.02 (8.8, 27.3)** | **18.9 (10.65, 27.01)** | **TRQ+**  **WMT** |  |
| **-2.18 (-4.37, -0.02)** | -2.88 (-6.25, 0.34) | -2.9 (-9.41, 3.57) | -2.01 (-6.89, 2.69) | **-20.92 (-27.56, -14.23)** | **WMT** |

Table3 League tables comparing interventions of CRP.

| **XBJ+**  **WMT** |  |  |  |  |  |
| --- | --- | --- | --- | --- | --- |
| **-**13.39 (-39.3, 12.88) | **SF+**  **WMT** |  |  |  |  |
| **24.8 (5.49, 43.78)** | **38.14 (7.3, 68.19)** | **SM+**  **WMT** |  |  |  |
| -8.47 (-24.28, 7.1) | 4.82 (-23.76, 32.92) | **-33.26 (-55.39, -11.18)** | **SQ+**  **WMT** |  |  |
| -12.34 (-35.32, 10.43) | 0.94 (-32.12, 34.13) | **-37.09 (-64.78, -9.49)** | -3.89 (-29.25, 21.53) | **TRQ+**  **WMT** |  |
| **-19.4 (-27.36, -11.63)** | -6.06 (-31.15, 18.67) | **-44.19 (-61.48, -26.73)** | -10.87 (-24.49, 2.65) | -7.02 (-28.52, 14.62) | **WMT** |

Table4 League tables comparing interventions of TNF-α.

| **XBJ+**  **WMT** |  |  |  |  |  |  |
| --- | --- | --- | --- | --- | --- | --- |
| 10.86 (-63.07, 85.39) | **SF+**  **WMT** |  |  |  |  |  |
| 7.5 (-49.8, 66.48) | -3.41 (-85.66, 80.08) | **SM+**  **WMT** |  |  |  |  |
| -35.35 (-98.37, 27.59) | -46.1 (-133.11, 40.34) | -42.83 (-116.86, 29.09) | **SQ+**  **WMT** |  |  |  |
| -44.68 (-119.57, 29.43) | -55.43 (-150.95, 39.67) | -52.2 (-136.18, 30.12) | -9.52 (-96.49, 77.99) | **HQ+**  **WMT** |  |  |
| -48.38 (-147.75, 51.16) | -59.09 (-174.82, 56.52) | -55.88 (-162.1, 50.13) | -12.83 (-122.37, 96.48) | -3.83 (-119.79, 112.47) | **TRQ+**  **WMT** |  |
| **-48.79 (-80.21, -17.34)** | -59.75 (-126.89, 7.01) | **-56.25 (-105.93, -8.21)** | -13.36 (-67.93, 41.38) | -4.03 (-71.46, 63.41) | -0.42 (-94.79, 93.4) | **WMT** |

Table5 League tables comparing interventions of WBC.

| **XBJ+**  **WMT** |  |  |  |  |  |
| --- | --- | --- | --- | --- | --- |
| -1.78 (-5.39, 1.84) | **SF+**  **WMT** |  |  |  |  |
| -0.43 (-4.42, 3.51) | 1.35 (-3.88, 6.56) | **SM+**  **WMT** |  |  |  |
| -0.04 (-2.61, 2.55) | 1.74 (-2.53, 5.98) | 0.4 (-4.16, 4.99) | **SQ+**  **WMT** |  |  |
| -2.32 (-5.77, 1.15) | -0.54 (-5.38, 4.32) | -1.88 (-7.02, 3.22) | -2.29 (-6.43, 1.85) | **TRQ+**  **WMT** |  |
| **-2.53 (-3.4, -1.64)** | -0.76 (-4.27, 2.77) | -2.11 (-5.94, 1.82) | **-2.5 (-4.92, -0.07)** | -0.21 (-3.55, 3.16) | **WMT** |

Table6 League tables comparing interventions of 28-day mortality.

| **XBJ+**  **WMT** |  |  |  |  |
| --- | --- | --- | --- | --- |
| 0.35 (-2.63, 3.32) | **SQ+**  **WMT** |  |  |  |
| 0.29 (-2.59, 3.22) | -0.06 (-4, 3.9) | **HQ+**  **WMT** |  |  |
| -0.34 (-1.25, 0.58) | -0.69 (-3.53, 2.14) | -0.63 (-3.42, 2.13) | **WMT** |  |
