# Supplementary material for: Comparative efficacy and safety of Chinese botanical drug injection in patients with sepsis: A systematic review and Bayesian network meta-analysis of randomized clinical trials
Source: PLoS One. 2026 Mar 24;21(3):e0343026. doi: 10.1371/journal.pone.0343026 (PMC13012499; doi:10.1371/journal.pone.0343026)
Supplement: S8 File — Judgment criteria and downgrading rationale applied for assessing the certainty of evidence using the GRADE framework. (DOCX) [file pone.0343026.s008.docx]

**Criterion for judgment of GRADE**

| Outcomes | No. | Risk of bias | Inconsistency | Indirectness | Imprecision | Publication bias | GRADE  evidence quality |
| --- | --- | --- | --- | --- | --- | --- | --- |
| APACHE Ⅱ score | 45 | The risk of bias is high, and the reasons include insufficient implementation of randomization and blinding. | The research results are inconsistent and the I² value is high, indicating a large degree of heterogeneity. | The research is directly targeted at the intervention and the population, and is consistent with the research objective. | The sample size is adequate, the confidence interval is narrow, and the results are precise. | There was no obvious publication bias and the funnel plot was symmetrical. | ⊕⊕⊕⊕○  Moderate quality |
| PCT | 21 | The risk of bias is high, and the reasons include insufficient implementation of randomization and blinding. | The research results are inconsistent and the I² value is high, indicating a large degree of heterogeneity. | The research is directly targeted at the intervention and the population, and is consistent with the research objective. | The sample size is adequate, the confidence interval is narrow, and the results are precise. | There is publication bias and the funnel plot is asymmetric. | ⊕⊕○○  Low quality |
| CRP | 34 | The risk of bias is high, and the reasons include insufficient implementation of randomization and blinding. | The research results are inconsistent and the I² value is high, indicating a large degree of heterogeneity. | The research is directly targeted at the intervention and the population, and is consistent with the research objective. | The sample size is adequate, the confidence interval is narrow, and the results are precise. | There is publication bias and the funnel plot is asymmetric. | ⊕⊕○○  Low quality |
| TNF-α | 28 | The risk of bias is high, and the reasons include insufficient implementation of randomization and blinding. | The research results are inconsistent and the I² value is high, indicating a large degree of heterogeneity. | The research is directly targeted at the intervention and the population, and is consistent with the research objective. | The sample size is adequate, the confidence interval is narrow, and the results are precise. | There is publication bias and the funnel plot is asymmetric. | ⊕⊕○○  Low quality |
| WBC | 26 | The risk of bias is high, and the reasons include insufficient implementation of randomization and blinding. | The research results are inconsistent and the I² value is high, indicating a large degree of heterogeneity. | The research is directly targeted at the intervention and the population, and is consistent with the research objective. | The sample size is adequate, the confidence interval is narrow, and the results are precise. | There is publication bias and the funnel plot is asymmetric. | ⊕⊕○○  Low quality |
| 28-day mortality | 16 | The risk of bias is high, and the reasons include insufficient implementation of randomization and blinding. | The research results are inconsistent and the I² value is high, indicating a large degree of heterogeneity. | The research is directly targeted at the intervention and the population, and is consistent with the research objective. | The sample size is adequate, the confidence interval is narrow, and the results are precise. | There was no obvious publication bias and the funnel plot was symmetrical. | ⊕⊕⊕⊕○  Moderate quality |
